# A novel broad spectrum venom metalloproteinase autoinhibitor in the rattlesnake *Crotalus atrox* evolved via a shift in paralog function

**DOI:** 10.1073/pnas.2214880119

**Published:** 2022-12-12

**Authors:** Fiona P. Ukken, Noah L. Dowell, Mamta Hajra, Sean B. Carroll

**Affiliations:** ^a^HHMI, University of Maryland – College Park, College Park, MD 20742; ^b^Department of Biology, University of Maryland – College Park, College Park, MD 20742

**Keywords:** evolution, gene family, snake venom, enzyme inhibition

## Abstract

The complexity of snake venom composition reflects adaptation to the diversity of prey and may be driven at times by a coevolutionary arms race between snakes and venom-resistant prey. However, many snakes are also resistant to their own venom due to serum-borne inhibitors of venom toxins, which raises the question of how snake autoinhibitors maintain their efficacy as venom proteins evolve. To investigate this potential three-way arms race among venom, prey, and autoinhibitors, we have identified and traced the evolutionary origin of serum inhibitors of snake venom metalloproteinases (SVMPs) in the Western Diamondback rattlesnake *Crotalus atrox* which possesses the largest known battery of SVMP genes among crotalids examined. We found that *C. atrox* expresses five members of a Fetuin A-related metalloproteinase inhibitor family but that one family member, FETUA-3, is the major SVMP inhibitor that binds to approximately 20 different *C. atrox* SVMPs and inhibits activities of all three SVMP classes. We show that the *fetua-3* gene arose deep within crotalid evolution before the origin of New World species but, surprisingly, *fetua-3* belongs to a different paralog group than previously identified SVMP inhibitors in Asian and South American crotalids. Conversely, the *C. atrox* FETUA-2 ortholog of previously characterized crotalid SVMP inhibitors shows limited activity against *C. atrox* SVMPs. These results reveal that there has been a functional evolutionary shift in the major SVMP inhibitor in the *C. atrox* lineage as the SVMP family expanded and diversified in the *Crotalus* lineage. This broad-spectrum inhibitor may be of potential therapeutic interest.

## Significance

This study investigates how rattlesnakes protect themselves from their own evolving venom. Venom toxins can be dangerous if they enter the snake’s tissues or circulatory system during feeding or digestion, and many venomous species have evolved autoresistance to their own toxins. Here, we identify a broad-spectrum serum inhibitor of rattlesnake metalloproteinases, a group of toxins that causes tissue destruction and hemorrhage. We show that this inhibitor is a different member of a protein family known to inhibit metalloproteinases in Asian and South American relatives of rattlesnakes. We suggest that changes in the number and diversity of rattlesnake metalloproteinases selected for the emergence of this broad-spectrum inhibitor, which may be potentially useful in snakebite treatment.

Innovation and coevolution play major roles in the evolution of biological diversity. Evolutionary innovations (novelties) enable organisms to adopt new lifestyles and exploit new niches and can trigger major radiations while coevolutionary interactions among organisms (e.g., predator–prey, plant–herbivore, and host–parasite) are thought to spur evolutionary innovation and drive diversification (1, 2). Thus, the genetic origins of evolutionary novelties and the mechanistic bases of coevolutionary interactions are of central interest in evolutionary biology.

Animal venoms in general and snake venoms in particular offer rich models for exploring the molecular bases of novelty and coevolution. Most snake venoms contain an arsenal of toxin proteins that facilitate the immobilization, killing, and initial digestion of prey (3). Resistance to specific venoms has evolved in both snake prey as well as animals that prey on snakes, and this resistance appears to be adaptations to predation pressures (reviewed in (4, 5, 6). In many instances, resistance is conveyed by specific serum proteins that inhibit the biological activity of major toxins such as snake venom metalloproteinases (SVMPs) (5, 7). For example, the Mexican ground squirrel (8), California ground squirrel (9, 10), and New Mexican rock squirrel (11) have evolved resistance to particular rattlesnake venoms via SVMP inhibitors (12).

The ability of prey to evolve venom resistance can trigger a coevolutionary arms race in which snake predators in turn evolve toxins or venom compositions that circumvent resistance, as has been shown, for example, in the case of California ground squirrels and the Northern Pacific rattlesnake (*C. oreganus*) (11, 13). Indeed, it is widely thought that the diversity of snake venom composition reflects adaptation to the diversity and susceptibility of prey in their diets (14, 15, 16, 17).

However, the potential for venom–prey coevolution raises yet another facet of possible coevolutionary pressure on venomous snakes that has not yet received much attention. Many venomous snakes are themselves resistant to their own venom (18, 19, 20, 21), reviewed in refs. 5 and 22, which has been proposed to protect the snakes from exposure to venom toxins during feeding and digestion (23). Autoresistance in many viperids has been shown to involve serum proteins that inhibit venom hemorrhagic and protease activities (24, 25, 26). The specific proteins responsible for venom resistance have been identified in a few crotalids such as *Protobothrops flavoviridis* (27, 28), *Gloydius blomhoffi* (29), and *Bothrops jararaca* (30) that each express a Fetuin-related cystatin-type metalloprotease inhibitor (named “serum factor” by Omari-Satoh 1972 (27)) that inhibits autologous SVMP activities.

The process of venom–prey coevolution raises the question of how snake autoinhibitors coevolve to maintain inhibition of evolving venoms? One possibility is that the diversification of venom proteins is accompanied by the expansion and diversification of autoinhibitors. Alternatively, autoinhibitors may maintain their inhibitory activity despite the diversification of toxin sequences and structures.

To explore this potential three-way arms race among venom, prey, and autoinhibitors, we have identified and traced the evolutionary origin of serum-borne SVMP inhibitors in the Western Diamondback rattlesnake *Crotalus atrox*. We selected this species because we have previously shown that it possesses by far the largest known battery of SVMP genes (30 loci) of any crotalid (31); other rattlesnake species studied possess 5 to 15 SVMP genes (31–33). We anticipated that the greater expansion and diversification of SVMPs in this lineage may have in turn influenced the evolution of the number and/or activity of potential SVMP inhibitors. We found, however, that the number of Fetuin A-related metalloproteinase inhibitor family members was only slightly greater in *Crotalus* than in other crotalid genera, and no greater in *C. atrox* than in other *Crotalus* species with smaller SVMP gene batteries. Rather, we discovered that the activities of two family members have shifted dramatically in the evolution of the *C. atrox* lineage. One family member FETUA-3 is the major, broad-spectrum inhibitor of venom SVMP activities in *C. atrox*, but its orthologs are not major inhibitors in other crotalid genera. Conversely, a second family member FETUA-2 exhibits limited activity against *C. atrox* SVMPs, but its orthologs are the major serum SVMP inhibitors in Asian and South American crotalid genera. We conclude that there has been an evolutionary shift in the major serum SVMP inhibitor as the SVMP family expanded and diversified in the *Crotalus* lineage. The broad activity of this inhibitor may be promising for the potential treatment of crotalid envenomation.

## Results

### *C. atrox* Sera Inhibits Venom Metalloproteinase Activity.

Metalloproteinases constitute about half of *C. atrox* venom protein by weight (34). Autoresistance to *Crotalus* venom has been attributed to proteins found in the animal’s serum (20), and antihemorrhagic factors that inhibit venom protease activity have been isolated from *C. atrox* serum (25, 26), but the specific proteins responsible have not been identified.

**Fig. 1.**
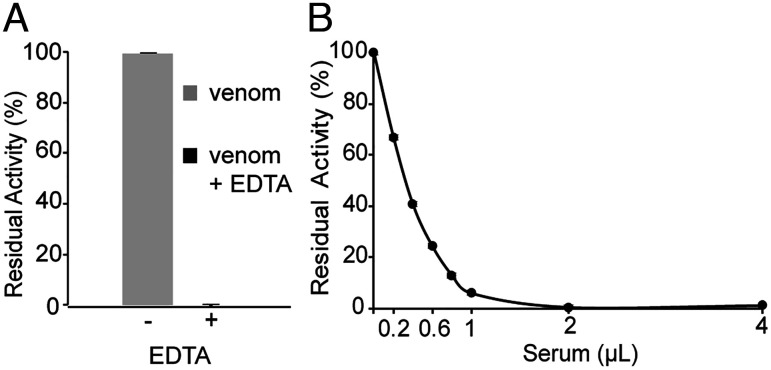
*C. atrox* venom contains metalloproteinase activity that is inhibited by *C. atrox* serum. (*A*) Ten micrograms (µg) of whole *C. atrox* venom preincubated in the presence (+) or absence (−) of 1 mM EDTA was assayed for proteolysis of a collagen substrate (Azocoll). The presence of EDTA in the reaction abolishes detectable metalloproteinase proteolysis of collagen. (*B*) Whole-venom proteolysis of collagen is inhibited by *C. atrox* serum. Error bars indicate standard deviation.

Collagenase activity is widely used as a proxy for the hemorrhagic activities of SVMPs (35). To identify and characterize proteins that confer resistance to metalloproteinase activity in the rattlesnake *C. atrox*, we used activity on a collagen-based substrate (Azocoll) as an initial assay for metalloproteinases and their inhibitors. Divalent cations such as Zn^2+^ and Ca^2+^ are critical for metalloproteinase function, and chelators such as EDTA inhibit their proteolytic activity (36). To test whether the activity of whole *C. atrox* venom on Azocoll was sensitive to EDTA, we added 1 millimolar (mM) EDTA and observed complete inhibition of the proteolytic activity, indicating that Azocoll was a suitable substrate for measuring the SVMP activity and its potential inhibition (Fig. 1*A*).

We tested the ability of increasing amounts of whole *C. atrox* sera to inhibit whole *C. atrox* venom proteolytic activity and observed dose-dependent inhibition. Just 2 microliters (µl) of sera inhibited the proteolytic activity of 10 micrograms (µg) of whole venom by 97 %, thus showing that *C. atrox* sera contains SVMP inhibitory activities (Fig. 1*B*).

### *C. atrox* Expresses Five Distinct Tandemly Arranged *fetuin-A* Genes.

In order to isolate potential serum inhibitors of SVMP activity, we first used a molecular homology strategy. Previous work has identified serum FETUA proteins from crotalid species including *G. blomhoffi* (37), *P. flavoviridis* (28), and *B. jararaca* (30) that inhibit SVMP and hemorrhagic activities to varying degrees. Because of the known activities of FETUA-type SVMP inhibitors activity in both Asian and South American genera, we focused our investigation on this class of potential inhibitors in *C. atrox* (a North American crotalid).

**Fig. 2.**
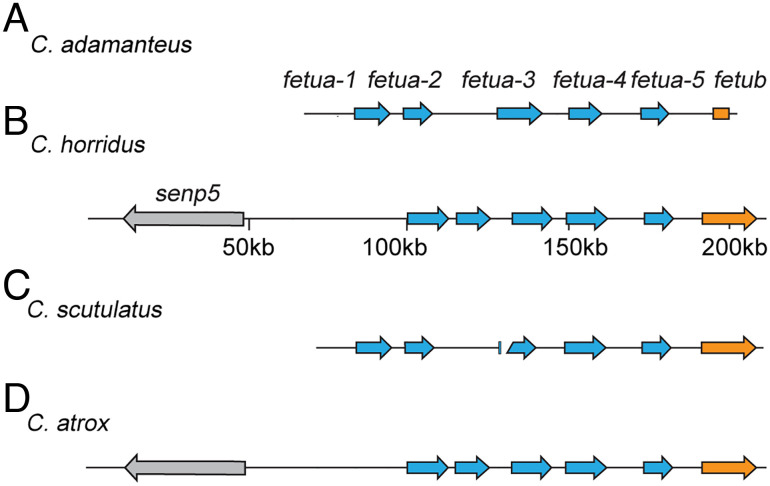
The *fetua* gene complex in *Crotalus* contains five loci. (*A*–*D*) The relative positions and orientations of *fetua* genes (blue arrows) in *C. adamanteus* (*A*), *C. horridus* (*B*), *C. scutulatus* (*C*), and *C. atrox* (*D*). The *C. adamanteus* genomic region contains five *fetua* genes. The *C. horridus fetua* locus shows that the five tandemly arranged *fetua* genes are flanked by the *senp5* and *fetub* genes. The *C. scutulatus fetua* locus shows a total of five *fetua* genes on two separate contigs. The reference-guided assembly of the *C. atrox fetua* gene complex also shows five *fetua* genes.

To isolate individual *C. atrox fetua* genes, we designed RT-PCR primers based on the sequence of Habu Serum Factor (HSF), a well-characterized FETUA family inhibitor from *P. flavoviridis*. A preliminary BLAST search of *C. atrox* tissue transcriptomes (liver, pancreas, and kidney) using the HSF sequences detected the most high-scoring hits in the liver, so we used *C. atrox* liver mRNA as a template for RT-PCR and cloned the amplified products into sequencing vectors. Sequencing these cDNA clones revealed three open reading frames (ORFs) encoding *fetua* genes. To determine whether these ORFs are encoded by separate genetic loci or might be potential isoforms, we screened a *C. atrox* bacterial artificial chromosome (BAC) library for *fetua* genes. This library has previously yielded sets of BAC clones that span the large phospholipase A2 and SVMP gene complexes (31, 38). However, we recovered only a single clone that spanned parts of just two *fetua* genes (apparently due to a gap in the BAC library coverage). Therefore, we endeavored to obtain genomic information from other *Crotalus* species including *C. adamanteus*, *C. horridus, and C. scutulatus* to assess the size of and to annotate the *Crotalus fetua* region and discovered that it consisted of a 5-gene complex (Fig. 2 *A*–*C* and *SI Appendix*, Fig. S1). We then identified the *C. atrox fetua* gene complex using a reference-guided consensus assembly with the *C. horridus fetua* complex as the reference (Fig. 2*D* and *SI Appendix*, Figs. S2 and S3).

**Fig. 3.**
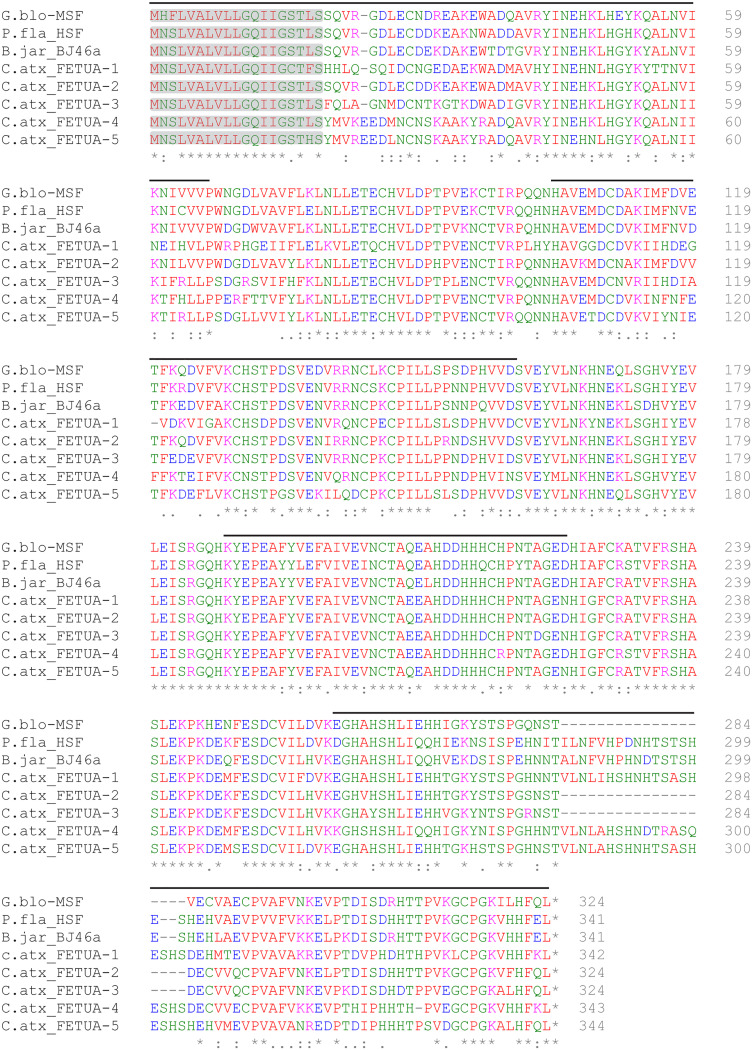
Alignment of *C. atrox* FETUA protein sequences with FETUA inhibitors from other Crotalinae species. *C. atrox* FETUA amino acid sequences were inferred from the nucleotide sequences of corresponding cDNA clones. A 19-residue deletion occurs in the seventh exon of *C. atrox* FETUA-2 and FETUA-3, in the same region as a 19-residue deletion in the *G. blomhoffi* MSF protein. Signal peptide regions are highlighted in gray and alternating exons are marked with black lines above the sequence. Conserved amino acid residues are indicated by asterisks (*). G. blo—*Gloydius blomhoffi*, P. fla—*Protobothrops flavoviridis*, B. jar—*Bothrops jararaca*, and C. atx—*Crotalus atrox.*

In *C. atrox*, as well as the other three *Crotalus* species we examined, each of the five tandemly arranged *fetua* gene loci was composed of seven exons spanning between 8 and 13 kilobase pairs (kb) and arranged in a head-to-tail fashion. Based on the genomic arrangement, we named the five genes *fetua-1, fetua-2, fetua-3, fetua-4, and fetua-5* (Fig. 2). In *C. atrox, fetua-1, fetua-4, and fetua-5* encode proteins of approximately 342 amino acids, while *fetua-2* and *fetua-3* encode proteins that are 19 amino acids shorter due to a deletion in the seventh exon (Fig. 3). A similar 19 amino acid deletion of the seventh exon is observed in the *Mamushi Serum Factor* (*MSF*) gene in *G. blomhoffi* (Fig. 3) (37).

To verify that all five genes are expressed in *C. atrox*, we used the genomic sequences to design primers to successfully amplify cDNAs corresponding to the two genes that were not recovered in the initial RT-PCR screen of *C. atrox* liver mRNA. The deduced amino acid sequences of the five encoded proteins exhibit strong similarities to one another and to the three previously identified serum SVMP inhibitors from *G. blomhoffi*, *P. flavoviridis*, and *B. jararaca* (Fig. 3).

### Only FETUA-3 is a Potent Inhibitor of *C. atrox* SVMP Activity.

Previous studies of other crotalids have reported no more than three genes in any species such as *G. blomhoffi* (29). The larger number of *fetua* genes in *C. atrox* and other *Crotalus* species is consistent with the possibility that this apparent expansion could be related to the expansion of SVMP loci (~30 genes in *C. atrox*) (31) and an accompanying requirement for a larger number of autoinhibitors.

To determine whether each *C. atrox* FETUA protein was able to inhibit SVMP activity, we expressed recombinant versions of each protein. To facilitate purification, each construct encoded an N-terminal His-FLAG tag. Because the incorporation of a hexahistidine tag can affect the activity of some recombinant proteins (39, 40), we first examined whether the presence of the His-FLAG tag might affect the activity of FETUA proteins. We performed enterokinase (EK) digestion to remove the His-FLAG tag and compared the activity of tagged and untagged/cleaved recombinant proteins in inhibiting *C. atrox* SVMP activity (*SI Appendix*, Fig. S4 *A* and *B*). We observed, for example, that the tag-free recombinant FETUA-3 protein was significantly more active in inhibiting whole-venom SVMP proteolytic activity than the tagged protein (*SI Appendix*, Fig. S4*C*). Therefore, we used untagged/cleaved recombinant FETUA proteins in all of our experiments (*SI Appendix*, Fig. S4*D*).

**Fig. 4.**
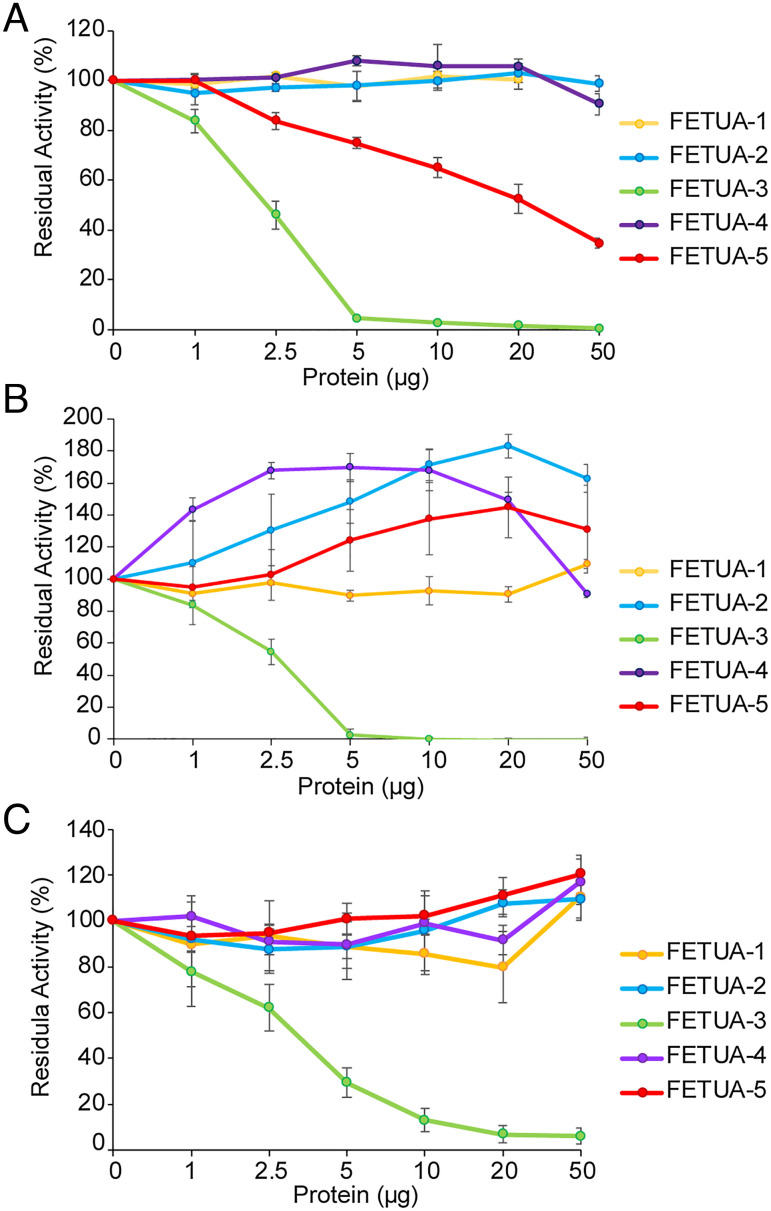
Inhibition of *C. atrox* proteolytic activity by FETUA proteins. (*A*) 50% inhibition of whole-venom collagen cleavage activity is achieved by ~2.5 µg of FETUA-3 (green line) while approximately of 20 µg of FETUA-5 protein is required to achieve 50% inhibition of whole-venom collagen cleavage activity (red line). Inhibition of whole-venom collagen cleavage activity by FETUA-1, FETUA-2, and FETUA-4 is undetectable. (*B*) MDC-4 (class III SVMP) proteolysis of collagen is completely inhibited by FETUA-3 while other FETUA proteins show no inhibition but enhance MDC-4 digestion of collagen. (*C*) FETUA-3 inhibits MPO-1 proteolysis of collagen. Percent residual activity refers to the enzyme activity remaining after incubation with inhibitors. In a 1-ml reaction volume, 1 µg of FETUA-3 is 0.027 µM, 4 µg of MDC-4 is 0.085 µM, and 4 µg of MPO is 1 0.157µM. Experiments were carried out at least three times, and error bars indicate standard deviation.

We examined the ability of each of the five recombinant FETUA proteins from *C. atrox* to inhibit the proteolytic activity of whole venom. We found that FETUA-1, FETUA-2, and FETUA-4 had no detectable inhibitory activity against whole venom on the Azocoll collagen substrate (Fig. 4*A*, yellow, blue, and purple lines). In contrast, FETUA-3 showed very strong inhibitory activity with 2.5 µg of protein inhibiting 50% and 5 µg of protein nearly completely inhibiting 10 µg of whole-venom collagenase activity (Fig. 4*A*, green line). FETUA-5 showed much lower SVMP inhibitory activity with ~50% inhibition observed with 20 µg of protein, respectively (Fig. 4*A*, red line).

Whole venom is a complex mixture and assays using it will fail to identify the FETUA activity against individual components. To further explore potential FETUA activities, we examined their ability to inhibit the activity of purified SVMP enzymes. There are three distinct classes of SVMPs (P-I, P-II, and P-III) that differ by the presence/absence of three distinct domains: all SVMPs contain a metalloproteinase domain; class P-III SVMPs also possess both disintegrin-like and C-terminal cysteine-rich domains; class P-II proteins lack the cysteine-rich domain but possess a disintegrin domain; and class P-I SVMPs possess only the metalloproteinase domain (41, 42). We refer to the P-I class as MPO proteins, the P-II class as MAD proteins, and the P-III class as MDC proteins (31). We purified MDC-4, MAD-3, and MPO-1 proteins, the most abundant class III, II, and I SVMPs in *C. atrox* venom, respectively (31) (*SI Appendix*, Fig. S5 and Supplementary Data 2). We found that FETUA-3 was able to completely inhibit MDC-4 activity (Fig. 4*B*, green line) and MPO-1 activity (Fig. 4*C*, green line) on the Azocoll substrate, but the other four FETUA proteins exhibited no inhibitory activity on either enzyme in this assay; rather, several FETUAs enhanced MDC-4 activity up to approximately 1.8 fold (Fig. 4*B*). MAD-3 has no activity on Azocoll, and therefore inhibition of MAD-3 could not be assessed on this substrate.

**Fig. 5.**
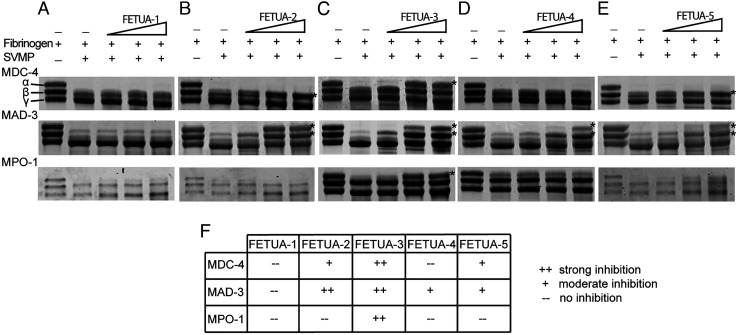
FETUA-3 inhibits all three classes of *C. atrox* SVMPs activities on fibrinogen. Inhibition of fibrinogenolytic activity of MDC-4, MAD-3, and MPO-1 by the five FETUA proteins was tested using bovine fibrinogen as the substrate. Effects of the FETUA inhibitors on SVMP fibrinogenolysis were assessed by observing cleavage of the α and β chains of bovine fibrinogen as decreased band intensity, the positions of the α-, β-, and γ-uncleaved forms are denoted in the control lane in the panel. (*A*–*E*) FETUA samples incubated with MDC-4, MAD-3, or MPO1, and fibrinogen, and analyzed by SDS-PAGE. Lane 1—fibrinogen alone lane 2—fibrinogen incubated with SVMP; and lanes 3–5—fibrinogen incubated with increasing amounts of FETUA (1, 2.5, and 5 μg). FETUA inhibition of SVMP fibrinogenolytic activity is indicated by asterisk (*). (*F*) A summary of FETUA inhibition of MDC-4, MAD-3, and MPO-1. FETUA-3 inhibits all three classes of SVMP. FETUA-2 and FETUA-5 show moderate inhibition of MDC-4 activity (β-chain cleavage) while FETUA-2, FETUA-4, and FETUA-5 inhibit cleavage of α and β chains by MAD-3. MPO-1 activity on fibrinogen is only neutralized by FETUA-3.

However, SVMPs can have different and multiple activities and are known to cleave other substrates such as fibrinogen. We next examined the ability of the five FETUA proteins to inhibit MDC-4, MPO-1, and MAD-3 activity on bovine fibrinogen. We observed that FETUA-3 was a strong inhibitor of all three SVMP enzyme classes on fibrinogen (Fig. 5*C*, summarized in Fig. 5*F*). We also detected some activity of other FETUAs: FETUA-2 showed comparable inhibition of MAD-3 as FETUA-3 but very weak inhibition of MDC-4 and no inhibition of MPO-1 (Fig. 5*B*); FETUA-4 showed modest inhibition of MAD-3 but no inhibition of MDC-4 or MPO-1(Fig. 5*D*); FETUA-5 showed modest inhibition of MAD-3 and MDC-4 but not MPO-1 (Fig. 5*E*); and FETUA-1 showed no activity against any of the three enzymes tested (Fig. 5*A*; all FETUA activities are summarized in Fig. 5*F*).

The potent activity of FETUA-3 and the narrow or absence of activity of the other FETUA family members raised the possibility that FETUA-3 might be the major inhibitor of *C. atrox* SVMP activity in *C. atrox* sera.

### *C. atrox* FETUA-3 Binds to at least 20 SVMPs.

**Fig. 6.**
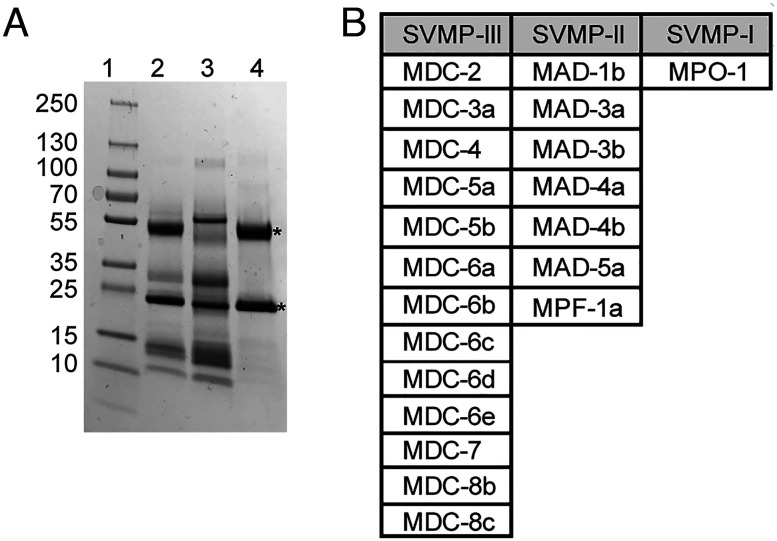
FETUA-3 binds approximately 21 SVMPs from all three classes. Affinity purification and identification of FETUA-3 interacting venom proteins from *C. atrox.* (*A*) SDS-PAGE of *C. atrox* venom purified on a FETUA-3 affinity column. Lane 1—protein standards, lane 2—whole-venom input, lane 3—flowthrough venom fraction from FETUA-3 affinity column, and lane 4—eluate. Asterisks (*) denote enriched proteins in eluate fraction that migrate at the expected sizes of class III and class I SVMPs. (*B*) Mass spectrometry of the FETUA-3 column eluate identified 21 SVMPs based on unique peptide spectra (*SI Appendix*, Supplementary Data 3). All three classes of SVMPs are represented in the eluate fraction.

Since FETUA-3 is highly effective at inhibiting whole-venom proteolytic activity and is able to inhibit representatives of each of the three classes of *C. atrox* SVMPs, it is possible that FETUA-3 may bind to additional SVMPs in *C. atrox* venom. To examine this possibility, we employed an affinity chromatography strategy using purified FETUA-3 as a ligand cross-linked to NHS-Trap agarose. We applied whole *C. atrox* venom to the FETUA-3 affinity column, washed the resin extensively, and eluted bound proteins with 0.1M glycine pH 2.7. The venom proteins eluted from the FETUA-3 affinity column were then separated by SDS-PAGE, individual bands were excised from the gel, and both the excised bands and the whole eluate were analyzed by mass spectrometry (Fig. 6*A*).

The proteins that bound to the FETUA-3 protein affinity resin were identified by unique peptide matches to individual SVMP proteins from a *C. atrox* venom/SVMP database (29), *SI Appendix*, Supplementary Data 3. We found that FETUA-3 bound to ~20 distinct SVMP proteins belonging to all three classes of SVMPs (Fig. 6*B*). This is nearly all SVMPs that are detectable and distinguishable by mass spectrometry (31). Of course, binding is not evidence of inhibition. However, the finding that FETUA-3 is capable of binding to nearly the entire spectrum of *C. atrox* SVMPs, coupled with the nearly complete inhibition of whole-venom activity by FETUA-3 and of representatives of three classes of SVMPs, indicates that FETUA-3 may be able to inhibit most *C. atrox* SVMPs.

We also tested the ability of FETUA-3 to inhibit the SVMP of whole venoms from other species including *C. horridus* (a relatively distant member of the genus) and *Sistrurus catenatus edwardsii* (a member of a separate rattlesnake genus). Both venoms were inhibited, with *C. horridus* activity inhibited in a similar degree to *C. atrox* venom (*SI Appendix*, Fig. S6*A*) and *S. catenatus edwardsii* (*SI Appendix*, Fig. S6*B*) to a slightly lesser degree. These results further suggest that FETUA-3 has the ability to bind to and inhibit a broad range of SVMPs.

FETUA-3 is the first FETUA protein from any snake species that has been found to bind to such a diverse array of SVMPs. This finding is also surprising in light of the expansion of the *C. atrox fetua* gene complex to five expressed genes, more than that are known to exist in other Crotalid genera (37). One might have expected that the larger number of FETUA-type inhibitors would be involved in inhibiting a larger array of SVMPs such as those that exist in *C. atrox*, but the other FETUAs show limited or no activity against SVMPs. FETUA proteins that lack SVMP inhibitory activities have also been reported in other crotalid species (29). This apparent paradox of possessing more FETUAs but of one protein exhibiting broad activity prompted us to examine the evolutionary relationship of FETUA-3 to previously described SVMP inhibitors.

### The FETUA-3 Protein Belongs to a Different Paralog Group than Previously Identified Crotalid SVMP Inhibitors.

**Fig. 7.**
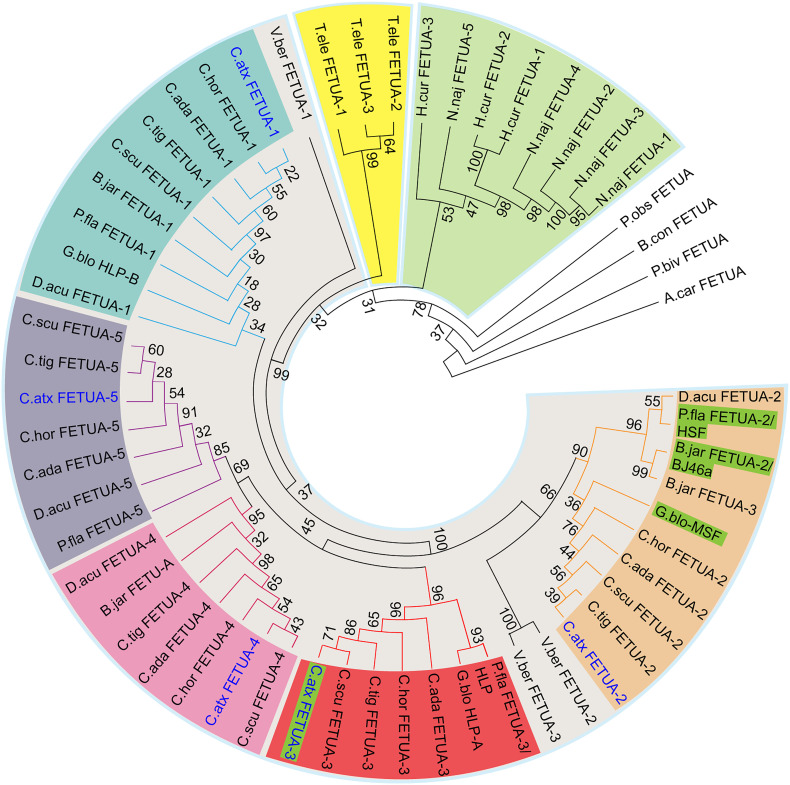
The FETUA-3 protein belongs to a different gene clade/paralog group than other crotalid SVMP inhibitors. A protein phylogeny of full-length FETUA proteins was constructed using sequences from available protein databases and hypothetical translations from our gene models and publicly available genomes. Taxa are represented by color-coded backgrounds: colubrids—yellow; elapids—green; and viperids—gray. Paralog genes in viperid species that are part of distinct, well-supported clades (bootstrap value > 80) are marked with similar colored nodes and arcs: FETUA-1—teal; FETUA-2—orange; FETUA-3—red, FETUA-4—pink; and FETUA-5—purple. *C. atrox* FETUA family proteins characterized in this study are in blue text, and FETUA proteins that are SVMP inhibitors are highlighted in green. Numbers at the nodes indicate the bootstrap values of 1,000 replicates. A complete list of genes, corresponding translations, and accession numbers can be found in *SI Appendix*, Supplementary Data 4.

To trace the evolutionary origin of the FETUA-3 and to determine its relationship to other previously identified Crotalid inhibitors—BJ46 (30), HSF (43), and MSF (37) and several Asian crotalid FETUA proteins that lack inhibitory activity—HLP, HLP-A, and HLP-B (29), we constructed phylogenies using predicted translations of FETUA proteins from published and publicly available genome data from four other *Crotalus* species, the South American crotalid *B. jararaca* and the Asian crotalids *D. acutus*, *P. flavoviridis*, and *G. blomhoffi* (Fig. 7 and *SI Appendix*, Fig. S7).

Crotalid FETUA proteins formed five distinct monophyletic groups (Fig. 7)*.* To our surprise, however, FETUA-3 nested within a strongly supported clade (BS = 96) that excluded all three previously described inhibitors from Asian and New World snakes HSF, MSF, and BJ46a, respectively (Fig. 7; bright red shading). Moreover, the FETUA-3 clade includes two proteins, HLP from *P. flavoviridis* and HLP-A from *G. blomhoffi* that have been shown to lack SVMP inhibitory activity (29). Furthermore, the *C. atrox* FETUA-2 protein, which has relatively limited SVMP inhibition activity, belongs to the clade that includes the inhibitors HSF, MSF, and BJ46a (Fig. 7; light orange shading). Thus, FETUA-3 is paralogous to known SVMP inhibitors and orthologous to previously characterized noninhibitors; conversely, the less active *C. atrox* FETUA-2 is orthologous to all previously identified inhibitors.

**Fig. 8.**
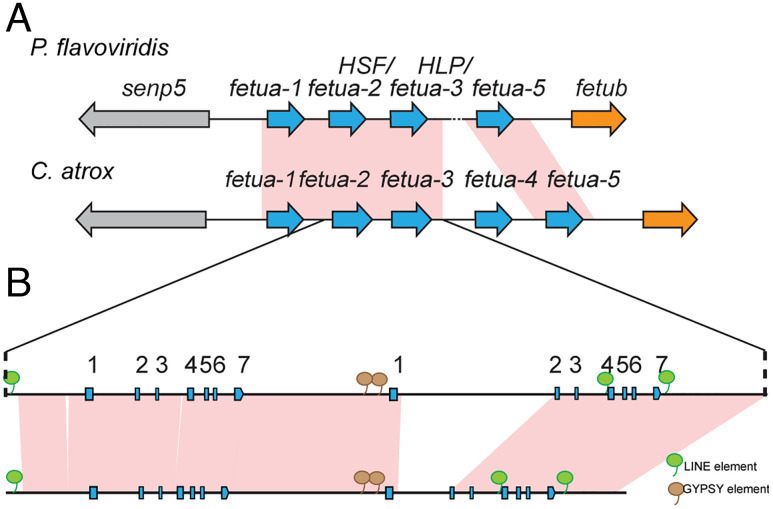
The synteny of *C. atrox* and *P. flavoviridis fetua-2* and *fetua-3* loci confirms functional shifts in the two paralogs. (*A*) Schematic showing synteny of the *fetua* gene complexes of *P. flavoviridis* and *C. atrox* (not to scale)*.* Syntenic regions are connected by pink (>90% identity) connecting blocks as determined by BLAST Z. A *fetua-4* gene is not detected in *P. flavoviridis* due to an ambiguous stretch of the nucleotide sequence (dotted line). The contiguous regions are indicated by solid lines. (*B*) A magnified view comparing the *fetua-2* and *fetua-3* gene loci in *P. flavoridis* and *C. atrox.* The exons are indicated by boxes, and arrows indicate the direction of transcription. Brown bubbles indicate the positions of shared Gypsy elements, while green bubbles indicate the positions of shared LINE elements as determined by the Repeatmasker program.

Because these inferences from protein phylogenies are unexpected, we wanted to obtain further evidence to confirm that we have correctly identified key orthologs between species. We annotated the *P. flavoviridis fetua* gene region and compared it with the *C. atrox fetua* gene complex for regions of synteny. Our analysis revealed extensive synteny between the two complexes (except for the absence of the *fetua-4* gene in *P. flavoviridis*) and supported our inference of orthology between the *P. flavoviridis* genes *fetua-2* (encoding the SVMP inhibitor HSF) and *fetua-3* (encoding the noninhibitory HLP protein) and the *C. atrox fetua-2* and *fetua-3* loci, respectively (Fig. 8). Based on the evolutionary relationships of the *Protobothrops*, *Gloydius*, and *Bothrops* genera, it is most parsimonious to infer that FETUA-2 was the major SVMP inhibitor in their most recent common ancestor. Therefore, we conclude that there has been a functional evolutionary shift among FETUA paralogs during the course of the evolution of the *C. atrox* lineage from its most recent common ancestor shared with *Bothrops*, *Protobothrops*, and *Gloydius* with the FETUA-3 paralog gaining function as a major inhibitor and the FETUA-2 paralog losing that role.

### *fetua* Gene Number Expanded Independently in the Viperid, Elapid, and Colubrid Lineages.

The conserved number of *fetua* genes in *Crotalus* irrespective of the SVMP gene content and the only slightly larger number of *Fetua* genes in *Crotalus* relative to other crotalids do not support a recent arms race driven by gene expansion and divergence. However, there remains the possibility that the f*etua* family expanded earlier in crotalid evolution and that the diversity of the gene family has been sufficient to maintain autoinhibition, for example, through functional paralog shifts as shown above for FETUA-3.

**Fig. 9.**
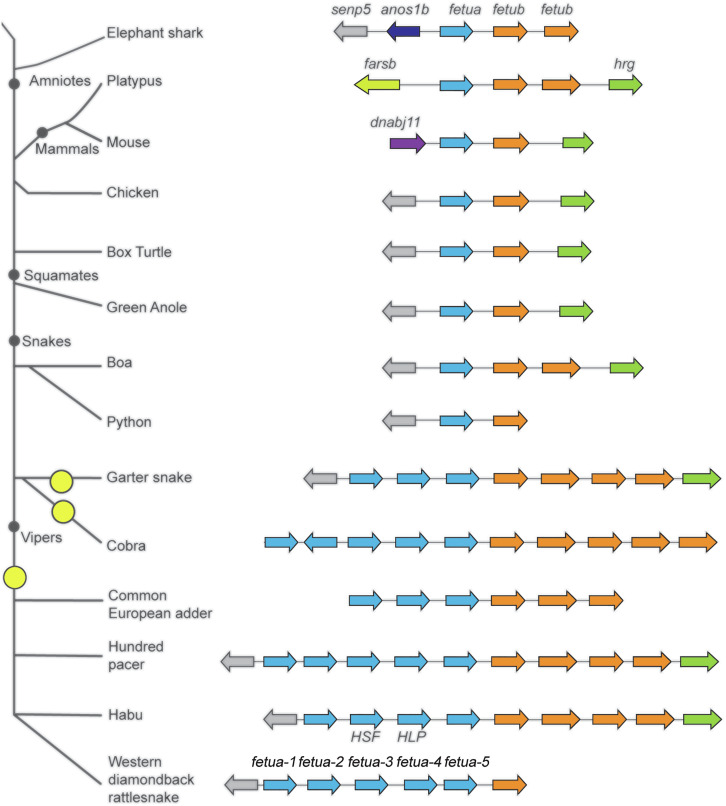
Independent expansions of the *fetua* gene complex occurred in three snake families. The organization of the *fetua* locus in fish, mammals, birds, and reptiles. Genes (colored arrows) within the same gene family have the same color (*fetua*—blue*, fetub*—orange*, senp5*—gray, and *hrg*—green). The *fetua* gene complex is flanked by *fetub* to the right and *senp5* to the left in all animals examined except for mammals and shark. Three independent expansions of the *fetua* locus occurred in snakes (indicated by yellow circles) and yielded multiple *fetua* genes in colubrids, elapids, and vipers. Elephant shark (*Callorhinchus milii*), mouse *(Mus musculus*), chicken (*Gallus gallus)*, box turtle (*Terrapene triunguis)*, green anole (*Anolis carolinensis*), boa (*Boa constrictor*), python (*Python bivittatus*), garter snake (*Thamnophis elegans*), cobra (*Naja naja*), common European adder (*Vipera berus*), hundred pacer (*Deinagkistrodon acutus*), Habu (*Protobothrops flavoviridis*), and western diamondback rattlesnake (*Crotalus atrox*).

To ascertain the evolutionary history of the *fetua* gene complex, we annotated genome sequences available from selected nonvenomous snakes (python and boa), various venomous snakes (elapid and viperid), and colubrid snakes (which include nonvenomous and venomous species) as well as from other amniotes. In all of the snake genomes analyzed where the flanking sequence was available, the *fetua* gene complex is flanked by the *fetub* and *senp5* loci as in *Crotalus* species, which provide useful landmarks for determining the extent of *fetua* gene expansion. This syntenic genomic arrangement is also conserved in lizards, turtles, and birds (Fig. 9).

We observed that both the python and boa genomes we examined contain just one *fetua* gene that is flanked by *fetub* and *senp5* genes (Fig. 9). The presence of one *fetua* gene is also the state in Western Rat Snake (*Pantherophis obsoletus)*, a colubrid (Fig. 7). However, we note that another colubrid, *Thamnophis elegans*, possesses three *fetua* genes (Figs. 7 and 9). A still greater number of five *fetua* genes was observed in an elapid, the Indian cobra (*Naja naja*) (Fig. 9).

Among viperids, we found at least three *fetua* genes in *Vipera berus.* (The locus was flanked by the *fetub* gene at the 3′ end of the complex, but the genomic sequence 5′ of the complex was not available) In Crotalinae, an examination of *D. acutus* revealed the presence of five *fetua* genes. In addition, analysis of *P. flavovirdis* revealed the presence of four *fetua* genes, including that encoding the inhibitor HSF (43). Furthermore, we were also able to detect at least four genes coding for FETUA proteins in *B. jararaca*, one of which encodes the inhibitor BJ46a.

The synteny of the *fetub* and *senp-5* loci that flank the *fetua* (also known as *alpha-2-HS-glycoprotein*, *AHSG*) locus in the amniote vertebrates examined suggests that *fetua* genes were derived from an ancestral *AHSG/fetua* gene. This single protein has been found in mammals to interact with a wide range of proteinases: matrix metalloproteinases, meprin metalloproteinases, and the cysteine proteases m-calpain, cathepsin L, and cathepsin V (reviewed in ref. 44). The expansion in the *fetua* gene number is a snake-specific feature. However, gene phylogenies reveal that this was not a single expansion process. Rather, we find that the five *Naja naja* genes belong to a separate clade from the viperid genes and have no known orthologs in other genera (Fig. 7; green shading). Similarly, the three *T. elegans* genes also belong to a distinct clade (Fig. 7; yellow shading). By contrast, all viperid *fetua* genes clustered separately from colubrid and elapid *fetua* family members (Fig. 7; gray shading). Based on the evolutionary relationships of these species and their *fetua* genes, we infer that the viperid/crotalid *fetua* gene complex expanded early in the evolution of the clade and that *fetua* gene duplications and expansion in elapids and colubrids occurred independently of those in viperids and one another.

## Discussion

This study was inspired by our recent discovery of the very large battery of SVMP genes in *C. atrox* and the hypothesis that the expansion and diversification of the SVMP gene complex selected for coevolutionary changes in the number or activity of serum SVMP autoinhibitors. We made three principal findings that bear on this issue, and they were unexpected to varying degrees. First, we did find a larger number of five *fetua* genes in *C. atrox* than the three loci that were known from other crotalid genera, but we determined that there was no recent expansion of the *fetua* gene family within *Crotalus* (we also found that other crotalids have at least four *fetua* genes). Second, of the five expressed *C. atrox* FETUA-type family members, we showed that only FETUA-3 is a potent, broad-spectrum SVMP inhibitor that binds to most *C. atrox* SVMPs and inhibits proteolytic activities of proteins from all three SVMP classes. Third, we discovered that there has been a functional evolutionary shift among FETUA paralogs during the divergence of crotalid genera, with the FETUA-3 paralog gaining function as a major inhibitor in *Crotalus* and the FETUA-2 paralog losing that role. These findings raise several questions concerning how and why a single but distinct FETUA protein evolved to act as the major SVMP inhibitor in *C. atrox* and whether the broad spectrum of FETUA-3 activity may be useful in the treatment of snakebites.

### Assessing the Arms Race Hypothesis for the Evolution of Crotalid SVMP Autoinhibitors.

There is a variety of well-studied examples of evolutionary arms races in which innovation in one species spurs coevolutionary adaptations in an ecologically interacting counterpart. Many such arms races involve species that produce toxins as defenses against predators or as weapons against prey (45). In such animals, however, it is also the case that the toxin producer must avoid self-intoxication. Thus, autoresistance is typically a part of the toxic or venomous phenotype and may need to evolve in response to and in step with the evolution of toxins and venoms.

We anticipated that the massive expansion and diversification of the SVMP gene family in *C. atrox* might have selected for a coevolutionary expansion of the *fetua*-encoded SVMP inhibitors that have previously been identified in other crotalid genera. To evaluate this possibility, it was necessary to identify the full genomic repertoire of *fetua* genes in *C. atrox* and to compare it with that of other crotalids (which had not been reported previously and was known only from cDNA sequences) (28, 30, 37). We identified five *fetua* genes in *C. atrox*, and the same number of loci in *C. adamanteus*, *C. scutulatus*, and *C. horridus*. Thus, there was no recent expansion of the *fetua* gene number that accompanied the expansion and diversification of SVMPs in the *C. atrox* lineage.

We also found that the *fetua* gene complexes in other crotalid genera are larger than that was known prior to this study, with four genes in *P. flavoviridis*, at least four genes in *B. jararaca*, and five genes in *D. acutus*. Furthermore, all five *D. acutus* and *C. atrox fetua* genes belong to the same five clades, revealing that the five-gene *fetua* complex was established early in a common ancestor and predated the divergence of the genera studied here. Therefore, we infer that *fetua* gene duplication has not accompanied any arms races that may have unfolded among SVMPs and prey during the divergence of these crotalids. Rather, it appears that four or five FETUA proteins have been sufficient to maintain autoresistance to SVMPs during crotalid evolution. This leads to the question of how these proteins maintain autoresistance in the face of SVMP expansion and diversification.

### One Major SVMP Inhibitor Binds to Most SVMPs and Inhibits all Three SVMP Classes.

There is a larger number of structurally and functionally diverse SVMPs than of FETUA proteins in most *Crotalus* species (31). One potential hypothesis to explain why there are five FETUAs and how they might maintain autoresistance is by inhibiting different subsets of SVMPs. But that is not what we found.

We tested the ability of the five *C. atrox* FETUA proteins to inhibit whole venom and purified class I, class II, and class III SVMP enzyme proteolysis of collagen and fibrinogen substrates. Only FETUA-3 was a potent inhibitor of all SVMP activities of whole venom, MDC-4, and MPO-1 action on collagen and of MDC-4, MAD-3, and MPO-1 on fibrinogen. The other four FETUAs largely exhibited weak or no detectable activity in most assays. Only FETUA-2 showed comparable activity to FETUA-3 in any assay, which was limited to the inhibition of MAD-3 action on fibrinogen. Notably, no protein besides FETUA-3 significantly inhibited the activity of the major SVMP MDC-4 (FETUA-2 exhibited only weak, partial inhibition of MDC-4 action on fibrinogen). And no protein except FETUA-3 exhibited inhibitory activity on representatives of all three classes of SVMPs. The broad action of FETUA-3 was further demonstrated by its ability when immobilized on an affinity column to bind to approximately twenty distinct *C. atrox* SVMPs spanning all three classes.

The apparently limited inhibitory activities of the other FETUAs seem paradoxical—why would an animal possess and express several proteins that appear to have relatively weak activity or narrow specificity? We can offer three possible explanations. First, it is not the case that the other four other FETUAs lacked activity altogether; we detected some activity for each protein (except FETUA-1) against at least one target in one assay. It is possible that these activities reflect some function that is sufficient for them to be maintained by natural selection. Second, it is also possible that we are not capturing the activities of some of these proteins in the limited in vitro assays used here. While these are standard assays in the field, we simply do not know the full range of proteins targeted by SVMPs in vivo. However, it is important to note that noninhibitory FETUAs have also been reported in other crotalids. Relatives of the major serum hemorrhagic inhibitors known from *P. flavoviridis* (HSF; designated FETUA-2 here) and *G. blomhoffi* (Mamushi serum factor, MSF; also, a FETUA-2 ortholog) have been isolated. Named, HLP and HLP-A, respectively, these FETUA-3 orthologs lack activity against various individual SVMPs (29) that are inhibited by HSF/FETUA-2 and MSF/FETUA-2 (37). And third, the other FETUAs may serve other physiological roles unrelated to venom resistance that have been maintained by selection.

What appears to be unprecedented among FETUA proteins examined thus far is *C. atrox* FETUA-3 broad specificity and strong inhibitory activity against all three classes of SVMPs. In comparison, the well-characterized FETUA-2 proteins from *P. flavoviridis* and *G. blomhoffi* fail to inhibit certain class II SVMPs (29). The ability of *C. atrox* FETUA-3 to interact with and inhibit all three classes of SVMPs suggests that FETUA-3 has evolved certain SVMP protein contacts that other FETUAs lack. Moreover, since other crotalid FETUA-3 proteins generally lack SVMP inhibitory activities, it must also be the case that FETUA-3 gained some key SVMP contacts in the *Crotalus* lineage not present in other FETUA-3 orthologs. The structural bases for the gains in FETUA-3 function will be important to determine.

### A Functional Evolutionary Shift between the FETUA-2 and FETUA-3 Paralogs.

In addition to the gains of function by the FETUA-3 protein, the very limited SVMP inhibitory activity of *C. atrox* FETUA-2 relative to its more broadly acting *P. flavoviridis, G. blomhoffi*, and *B. jararaca* orthologs reveals that FETUA-2 has lost its role as a major SVMP inhibitor in the evolution of the *Crotalus* lineage. This evolutionary shift in functions between the FETUA-2 and FETUA-3 paralogs was entirely unexpected.

Before seeking an explanation for this shift, our first concern was that we had correctly identified *fetua* orthologs and paralogs among crotalids. The grouping of the five *fetua* genes into five well-supported, distinct clades suggested that the evolution of the group was not remarkable, but we wanted to be sure that no genetic process had occurred that could confound gene identification. For example, if gene conversion had occurred between *fetua-2* and *fetua-3*, that could contribute to changes in gene function and alter gene phylogenies. However, one signature of gene conversion is that the two gene paralogs would be more similar to one another within a species than they are to their orthologs in other species. We do not observe such evidence in our gene trees. Moreover, we observe collinearity between the *P. flavoviridis* and *C. atrox fetua* gene complexes and their respective *fetua-2* and *fetua-3* loci. Taken together, these data suggest that changes in FETUA-2 and FETUA-3 functions evolved through ordinary mutation.

The question of why these two proteins’ roles changed is more difficult to discern. The fate of most members of multigene families is to functionally diverge, either through subfunctionalization of an ancestral gene, or neofunctionalization of individual paralogs. The evolutionary stability of members of a gene family, such as the *fetua* complex in crotalids explored here, would be expected to reflect selection to preserve their functions. Functional evolutionary shifts among paralogs, where one paralog takes on a function of another paralog, are relatively scarce in the literature, but we note that functional interconversions among paralogs are known. For example, among the plant cytochrome P450 (CYP) enzymes, different CYPs from the same family have independently evolved the same activity to adapt to similar environmental conditions (reviewed in ref. 46).

It is reasonable to infer that changes in FETUA paralog functions similarly reflect changes in selective conditions. Since the FETUA-2 function as a major SVMP inhibitor is shared among Asian and South American genera, one plausible scenario is that some differences in the SVMP composition in the *Crotalus* lineage selected for changes in autoinhibitor activity. One difference we note among the reported activities of the P. flavoviridis and *G. blomhoffi* FETUA-2 proteins and *C. atrox* FETUA-3 is that the Asian crotalid proteins appear not to inhibit some class II SVMPs (MADs) (28, 29, 37), whereas the *C. atrox* protein inhibits the MAD-3 class II SVMP and binds to at least seven additional class II SVMPs. *C. atrox* FETUA-2 also inhibits MAD-3. We suggest that changes in the number and diversity of class II SVMPs in the *Crotalus* lineage may have selected for changes in FETUA-2 and FETUA-3 activities that shifted their respective roles. Exploration of this hypothesis will require comparative structural analyses of FETUA inhibitor interactions with different SVMPs; detailed interaction data have only recently been obtained for one SVMP inhibitor—the *B. jararaca* SVMP inhibitor BJ46a and the SVMP jararhagin (47).

### The Potential Use of FETUA-3 in the Treatment of Snakebite.

Since the late 19th century, the treatment of venomous snakebite has entailed the administration of antivenoms composed of serum, serum fractions, or purified antibody preparations from animals (typically horses or sheep) hyperimmunized with whole snake venoms (48). The antibodies in antivenoms consist of populations of polyclonal antibodies that bind to various venom proteins and act by neutralizing and/or clearing venom toxins from circulation. Because of the nature of their means of production, it is not possible to control the potency of antivenoms against specific toxins. The ability of FETUA-3 to bind to and inhibit a wide range of SVMPs raises the possibility of incorporating it as a component of antivenom formulations that could enhance antivenom potency against SVMPs and their myriad clinical effects.

## Materials and Methods

### Venom, Serum Collection and Processing.

Lyophilized *C. atrox* (pool of ~200 snakes) and *C. horridus* venoms were purchased from Kentucky Reptile Zoo. *Sistrurus catenatus edwardsii venom and C. atrox* serum were purchased from the NNTRC, Texas A&M University, Kingsville. Venom stock solution was made by dissolving venom at 10 mg/mL in 20 mM Tris pH 8.0, 1 mM CaCl_2_. The solution was filtered through a 0.2 µm Durapore syringe filter and stored at −80 °C until use.

### RT-PCR and Cloning of *fetua* cDNAs.

Total RNA extracted from *C. atrox* liver samples with the Qiagen RNEasy kit was used to generate cDNA (SuperScript IV First-Strand Synthesis System) according to the manufacturer’s protocol. Specific cDNA sequences were amplified using Q5 polymerase (New England Biolabs) and the published Habu Serum Factor (HSF) primer sequence (37). This resulted initially in the isolation of a *C. atrox* FETUA-3 cDNA sequence encoding the *P. flavoviridis* C-terminal amino acid sequence KVHHFK instead of KALHFQ at amino acids 317 to 322. We later isolated full-length cDNAs encoding FETUA-1, FETUA-3, and FETUA-5 using oligo-dT primers. Due to the high degree of similarity between the *fetua* genes, additional primers were required for FETUA-2 and FETUA-4 sequences, and both gene sequences were recovered as two overlapping clones. For a list of primers used refer to *SI Appendix*, Supplementary Data 1A.

#### Crotalus *fetua* genomic region.

The *C. adamanteus fetua* locus was constructed from BAC libraries, while *C. scutulatus* and *C. horridus fetua* loci were determined from whole-genome sequencing data. The *C. atrox fetua* locus was determined by a reference-guided assembly of whole-genome shotgun sequencing reads to *C. horridus fetua* genomic region. Refer to *SI Appendix*, Supplementary Data for further details.

#### *C. atrox* liver transcriptome and translation.

The *C. atrox* liver RNA isolation, library preparation, sequencing, read processing, and transcript assembly were performed as described previously for venom gland transcriptomes (38). The 201589 assembled liver transcripts were translated using the Trinity helper script (Transdecoder.longestOrf) to obtain 113,718 liver-expressed peptides (49).

### Recombinant FETUA Protein Expression and Purification.

#### Generation of anti-FETUA-3 antibodies.

A DNA fragment encoding amino acids 78 to 268 of FETUA-3 was PCR amplified and cloned into the GST-tag expression vector pGEX-6P1. To express GST-cASF-B1, Rosetta-gami™ 2(DE3)pLacI (Agilent Technologies) cells were transformed with the recombinant construct. GST-FETUA-3 expression was achieved by induction with 1 mM IPTG. Since most of the protein was insoluble, we resorted to isolation of inclusion bodies (50). The recombinant protein containing inclusion body suspension was used to immunize two New Zealand white rabbits (UPFRL). Sera were tested for immunoreactivity on Western Blots, and antibodies were affinity purified on His-FLAG-FETUA-3 cross-linked to Aminolink Plus Coupling Resin (Thermo Scientific catalog # 20501).

#### Gene syntheses and plasmid construction.

The sequences coding for *C. atrox* FETUA proteins were based on sequences from RT-PCR. The endogenous signal peptide sequence (1 to 19 amino acids) was replaced with a sequence optimized for protein expression (MGWSCIILFLVATATGVHS) in mammalian cell lines. Additionally, a hexahistidine (6×His)-encoding sequence tag and a FLAG tag were inserted between the signal peptide and the remaining protein sequence (amino acids 20 to stop codon). The synthetized nucleotides were cloned into the mammalian expression vector pcDNA3.4 (Invitrogen, Carlsbad, CA, USA). All constructs were prepared commercially (GenScript, Piscataway, NJ, USA).

#### Transfection, protein production, and purification.

The recombinant FETUA protein expression plasmids were transiently transfected into either HD 293F/Expi 293F cells or HD-CHO-S cells, and FLAG-tag purified recombinant proteins were generated commercially (GenScript). The purified proteins were dialyzed against EK buffer (20 mM Tris, pH 8.0, 50 mM NaCl, and 2 mM CaCl_2_) and digested with EK light chain (New England Biolabs, P8070L) at a concentration of 0.2 U/µg of recombinant protein. The reaction mix was incubated at 25 °C for 16 h, following which EK was removed from the reaction mix using an EK Removal kit (Sigma PRKE). The concentrated tag-free recombinant proteins were dialyzed with either Tris-buffered saline (TBS) or phosphate-buffered saline and stored at −80 °C.

#### SDS-PAGE and Western blotting.

Protein samples were resolved by SDS-PAGE using 12% or 15% gels for 30 min at 200 V (Mini-Protean Cell; Bio-Rad), electrotransferred (18V for 30min, Trans-Blot® SD Semi-Dry Transfer or 7 min on Trans-Blot Turbo Transfer System, Bio-Rad) onto Immobilon-FL PVDF membranes (EMD Millipore), blocked with Odyssey® Blocking Buffer (TBS) (Li-Cor), and then incubated with the appropriate primary antibody (mouse anti-FLAG® M2 (1:3,000, Sigma) or rabbit anti-FETUA-3 (1:10,000)). Membranes were washed with TBS-Tween-20 followed by incubation with the appropriate Alexa Fluor™ 680/790-conjugated secondary antibody (1:15,000) in TBS-Tween-20. After further washing with TBS-Tween-20, the membranes were scanned using an Odyssey® CLx Imaging system. (Li-Cor).

### Affinity Purification of SVMPs.

We used recombinant FETUA-3 protein generated from *C. atrox* cDNA with the Habu primer that introduced Habu C-terminal amino acid substitutions (*P. flavoviridis*: KVHHFK: *C. atrox*: KALHFQ 317-322 aa) for the generation of an affinity column. The C. atrox FETUA-3 with Habu residues 317 to 322 is indistinguishable from the native C. atrox FETUA-3 protein with respect to inhibition of purified SVMPs or whole-venom digestion of Azocoll and fibrinogen substrates. Four milligrams of FETUA-3 was buffer exchanged into coupling buffer (0.2 M NaHCO_3_, 0.5 M NaCl, pH 8.3) and covalently bound to a 1 ml NHS-Trap HP column (GE) using ÄKTA prime and the manufacturer’s protocol. Two milligrams of lyophilized crude venom was resuspended in 20 mM Tris-HCl pH 7.4, 20 mM CaCl_2_, syringe filtered to remove particulate matter, and incubated on the FETUA-3-NHS-trap HP column for 1 h at 37 °C. After incubation, the column was connected to ÄKTA prime plus and washed with 20 mM Tris-HCl pH 7.4 and 20 mM CaCl_2_ followed by 20 mM Tris-HCl pH 7.4, 20 mM CaCl_2_, and 500 mM NaCl, 0.1% Tween-20 before eluting with 0.1 M glycine pH 2.7. The eluate fractions were collected in a tube containing 1/10 eluate volume of 1 M Tris pH 9. The pH-neutralized fractions were pooled and dialyzed against 20 mM Tris, pH 8, 1 mM CaCl_2_, concentrated, and stored at −80 °C and/or analyzed on 8 to 16% gradient SDS-PAGE gels with Coomassie staining. The eluate along with individually excised bands from gel was submitted for mass spectrometry analysis. For details, see *SI Appendix*, Supplementary Data Material and Methods.

### Purification of MDC-4, MPO-1, and MAD3 from *C. atrox* Venom.

The metalloproteinases used in the assays were purified from whole venom using ion exchange and size exclusion chromatography methods. For detailed description, see *SI Appendix*, Supplementary Data Methods.

### Proteolytic Assays.

#### Azocoll assay.

The Azocoll substrate was suspended at 5 mg/ml in 20 mM Tris-HCl pH 8.0. Working dilutions of venom was prepared from venom stock solution in 20 mM Tris-HCl pH 8.0 at 1 mg/ml. Ten micrograms of venom was incubated with increasing amounts of inhibitor candidates in 50 µl microliters and incubated at 37 °C for 1 h. The substrate (950 µl) was added to the enzyme–inhibitor solution and incubated at 37 °C for 60 min with agitation. Following incubation, samples were centrifuged at 15,000 × g (or RPM) for 2 min, the supernatant was collected, and absorbance was measured at 520 nanometers. All assays were performed in triplicate.

#### Fibrinogenolytic assay.

We used the method as described in (51) with modifications. FETUA proteins were incubated with one microgram of MDC-4, MAD-3, or MPO-1 at 37 °C for how long. Bovine fibrinogen (50 µl at 1 mg/ml, Sigma, catalog # 341573) in 50 mM Tris–HCl, pH 7.6, 150 mM NaCl was incubated with the enzyme–inhibitor mixture at 37 °C for 20 min. For MPO-1, the incubation time after the addition of the substrate was 60 min. The samples were then loaded on 8 to 16% gradient SDS–PAGE gels and analyzed by Coomassie staining.

### Phylogenetic Analysis.

Sequences used in our protein phylogenies were deduced from hypothetical translation of assembled genomic regions or genomic regions from NCBI databases (see the full list of sequences and accession numbers in *SI Appendix*, Supplementary Data 4). To construct protein phylogenies, we used MEGA software (v.11.0.11) (52) First, we performed multiple protein sequence alignment using MUSCLE feature (53) in the software. Next, we selected the best substitution model and built maximum likelihood trees using the Jones–Taylor–Thornton substitution model (54). Node support for the consensus tree was calculated by Bootstrap analysis on 1000 replicates trees.

Synteny between *P. flavoviridis* and *C. atrox* at the *fetua* loci was determined using the (B)LastZ algorithm (55) in GEvo feature at COGE (https://genomevolution.org/coge/GEvo.pl) (56). Transposable elements were identified using the Repeatmasker web server with the cross_match search engine run in slow mode (A.F.A. Smit, R. Hubley, and P. Green, unpublished data. Version: open-4.0.5, RMLib: 20140131, Dfam: 1.3).

## Supplementary Material

Appendix 01 (PDF)

## Data Availability

Gene sequences data have been deposited in [Genbank] (OP223730–OP223733, OP566391, OP440564,  and OP616715–OP616719). All study data are included in the article and/or *SI Appendix*.
