# Supplementary material for: A novel broad spectrum venom metalloproteinase autoinhibitor in the rattlesnake Crotalus atrox evolved via a shift in paralog function
Source: Proc Natl Acad Sci U S A. 2022 Dec 12;119(51):e2214880119. doi: 10.1073/pnas.2214880119 (PMC9907073; doi:10.1073/pnas.2214880119)
Supplement: Supplementary file 1 — Appendix 01 (PDF) [file pnas.2214880119.sapp.pdf]

Supporting Information for

A novel broad spectrum venom metalloproteinase auto-inhibitor in the rattlesnake *Crotalus atrox* evolved via a shift in paralog function

Fiona P. Ukken[1], Noah L. Dowell[1], Mamta Hajra[1] , and Sean B. Carroll[1]

Correspondence and request for materials should be addressed to Sean B. Carroll
Email: sbcarrol@umd.edu.

This PDF file includes:

Supporting text
Figures S1 to S7
Tables S1 to S4
SI References

Supplementary Methods

Reference-guided assembly of the *fetua* genomic region

Pools of clones from a previously constructed whole genome BAC library (5 -7x coverage, average insert size of 80 - 150 kb) for *C. adamanteus* were PCR screened with *fetua-3* primers (Supplementary data 1B) to identify clones positive for the *fetua-3* genomic sequence (1). The University of Michigan DNA sequencing core prepared the Pacific Biosciences sequencing libraries using ten micrograms of BAC DNA according to the standard protocol with a size selection of large (> 10000 bp) DNA fragments. The sequencing library for each single BAC clone was sequenced in a single SMRT cell. The raw reads were assembled using the accuracy optimized HGAP2 (Hierarchical Genome Assembly Protocol) algorithm (2).

The *C. horridus* and *C. scutulatus* draft genome assemblies have been described previously (3). The *fetua*-3 gene region was identified by LAST alignment of *C. atrox fetua* transcripts to *C. horridus* whole genome (4). Manual annotation of the *fetua* genes on the contig were combined with the crude gene coordinates from transcript alignments and with the inspection of hit coordinates for single exon BLASTs to produce refined gene models (5)

The *C. atrox fetua* gene complex was identified by alignment (bwa men) of processed (adapter trimmed, overlap merged) whole genome shotgun sequencing reads (~60X genome coverage) to the *C. horridus fetua* genomic region (6). Aligned reads were filtered using quality score (samtools -q 20), concatenated (samtools cat), sorted (samtools sort) and used to call nucleotide variants (samtools mpileup -0u -f horridus_fetA.fasta atrox_aligned_sort.bam | bcftools call -mv -0z -o atrox_fetA_region_) (7).

The variants were normalized (bcftools norm), filtered by removing adjacent variants within 5 basepairs of each other (bcftools filter —IndelGap 5) and indexed (bcftools index). The indexed variant file was then used to call a reference-based *C. atrox fetua* consensus genomic sequence (bcftools consensus) (8). Read coverage and gaps in coverage near the *fetua* gene loci was visualized using Gviz (9)

Purification of metalloproteinases from venom

MDC-4 purification

The purification of MDC4 from *C. atrox* venom followed the protocol of Williams et al. (10) with the following modifications, 400 mg of *C. atrox* venom was resuspended in 5 mL of 20 mM Tris-HCl buffer (pH 7.6), filtered through a 0.2 micron (µm).

Durapore syringe filter and applied to a 5 mL HiTrap Q XL Sepharose anion exchange column. Column was washed with 20 mM Tris-HCl buffer (pH 7.6) until UV returned to baseline. Protein elution was performed at a rate of 5 ml/min using 20 mM Tris-HCl (pH 7.6), 1 M NaCl gradient (up to 60%) by an ÄKTA FPLC system (GE Healthcare, UK) over 120 ml as 2.5 ml fractions. The collected fractions were run on SDS-PAGE gels, analyzed by Coomassie staining and fractions with the protein of interest (strong band migrating at ~ 50 kDa) were pooled. The pooled fractions were concentrated using a 10,000 MWCO Amicon Ultra -15 centrifugal filter and applied to a size exclusion chromatography column (HiLoad 16/600 Superdex 75 pg). Protein elution was performed at a rate of 1 ml/min using 20 mM Tris-HCl (pH 7.6) with1 ml fractions collected. The fractions were analyzed by SDS-PAGE and Coomassie staining and those containing strong band at ~50 kDa (MDC-4 and other expressed MDCs) were pooled and concentrated before a second run on the same size exclusion column. Fractions

containing the pure protein were pooled, concentrated, and stored as aliquots at -80˚C until further use (Fig S3 C). Protein estimation was performed using Quick Start Bradford 1x Dye Reagent (Bio-Rad) using bovine serum albumin (Thermo Scientific catalog # 23208,) as a standard.

MAD-3 and MPO-1 purification

C. atrox MAD-3 and MPO-1 were purified in a single protocol that used different buffer pH to separate MPO-1 (~24.7 kDa) from proteolytically processed MAD-3 (~19 kDa without disintegrin domain) (Fig S3 B). *C. atrox* venom (400 mg) was resuspended in 5 ml of 20 mM Bis-Tris buffer (pH 6.5),filtered through a 0.2 µm Durapore syringe filter and loaded onto a 5 ml HiTrap SP Sepharose cation exchange column. Protein elution was performed at a rate of 5 ml/min using 20 mM Bis-Tris buffer (pH 6.5), 1 M NaCl gradient (up to 60%) by an ÄKTA purifier system (GE Healthcare, UK) over 120 mL as 2.5ml fractions. The collected fractions were run on SDS-PAGE gels, analyzed by Coomassie staining and fractions with the protein of interest were pooled. The flow-through fraction contained MAD-3 while MPO-1 was present in gradient elution fractions. The flow-through fraction was concentrated using 10,000 MWCO Amicon Ultra -15 centrifugal filter and dialyzed against 20 mM Tris-HCl (pH 7.6) while the MPO-1 containing fractions were dialyzed against 20 mM Piperazine (pH 5.18).

For MAD-3 purification the pooled, concentrated flow-through fractions were loaded onto a 5 ml HiTrap Q XL Sepharose anion exchange column and eluted over a salt gradient (up to 60%) as in MDC-4 purification. Fractions containing MAD-3 were pooled, concentrated in a 10,000 MWCO centrifugal filter and dialyzed against 20 mM

Tris-HCl (pH 7.6), 300 mM NaCl before loading on to two size exclusion chromatography columns connected in tandem (HiLoad 16/600 Superdex 75 pg). Elution was performed at 0.5 ml/min and collected as 2.5 ml fractions and those containing protein of interest was pooled and concentrated (Fig. S3C).

The MPO-1 containing fraction (in 20mM Piperazine (pH 5.1)) was concentrated and the sample (5 mL) was applied to a 5 mL HiTrap SP Sepharose cation exchange column equilibrated in the same buffer. The column was washed with 20mM Piperazine (pH 5.1) until no protein was detected by the UV detector and eluted by a linear concentration gradient (0 to 60%) of 20 mM Piperazine (pH 5.1), 1 M NaCl under a flow rate of 0.5 ml/min and temperature of 4 °C. The eluted MPO-1 fractions were dialyzed against 1X PBS concentrated to a 1 ml sample volume and loaded onto two HiLoad 16/600 Superdex 75 pg columns connected in tandem. Protein elution was performed at a rate of 1 ml/min using PBS (pH 7.2) and 2.5 ml fractions were collected. The fractions were analyzed by SDS-PAGE and Coomassie staining and those containing MPO-1 were pooled and concentrated (10,000 MWCO) and stored as aliquots at -80˚C until further use. Protein estimation was performed using Quick Start Bradford 1x Dye Reagent (Bio-Rad) and bovine serum albumin (Thermo Scientific, catalog # 23208) as standards.

Mass Spectrometry

Enzymatic "In Gel" Digestion

Coomassie R-250 stained gel pieces were de-stained (50 % methanol (MeOH), 50% water (H_20), 100 mM acetic acid (NH_4HCO_3)) completely, dehydrated (50% acetonitrile (ACN), 50% H_20, 25 mM NH_4HCO_3) for five minutes and then incubated for 30 seconds in 100%

ACN. Next, the samples were dried in a Speed-Vac for one minute, reduced in 25 mM DTT (Dithiotreitol in 25mM NH_4HCO_3) for 15 minutes at 56°C, alkylated with 55 mM CAA (Chloroacetamide in 25 mM NH_4HCO_3) in darkness at room temperature for 15 minutes, washed once in H_2O, dehydrated (50% ACN, 50% H_20, 25 mM NH_4HCO_3) for two minutes in then incubated for 30 seconds in 100% ACN. The samples were dried again and rehydrated with 20 µl of trypsin solution with 0.01% ProteaseMAX™ surfactant (10 ng/µl *Trypsin* from Promega Corp. in 25 mM NH_4HCO_3, 0.01% w/v of ProteaseMAX™ from Promega Corp.), let stand for 2 minutes at room temperature then an additional 30 µl of overlay solution (25 mM NH_4HCO_3, 0.01% w/v of ProteaseMAX™) was added to keep gel pieces immersed throughout the digestion (three hours at 42°C). Peptides generated from digestion were transferred to a new tube and acidified with 2.5% TFA (trifluoroacetic Acid) to a 0.3% final concentration. Gel pieces were extracted further with 70 % ACN, 29.25% H_2O, 0.75% TFA for ten minutes while vortexing and solutions combined and dried completely in a Speed-Vac (~15 minutes). Extracted peptides were solubilized in 30 µl of 0.05% TFA. Degraded ProteaseMAX™ was removed via centrifugation (max speed, ten minutes) and the peptides solid-phase extracted (*ZipTip® C18* pipette tips from MilliporeSigma) according to the manufacturer's protocol. Peptides were eluted off the C18 SPE column with five microliters of 70% ACN, 30% H_2O, 0.1%TFA, dried to completion, then resolubilized in 30 µl total volume with 0.1% formic acid, and two microliters was loaded on the instrument.

NanoLC-MS/MS

Peptides were analyzed by NanoLC-MS/MS using the Agilent 1100 nanoflow system (Agilent) connected to a hybrid linear ion trap-orbitrap mass spectrometer (LTQ-Orbitrap Elite™, Thermo Fisher Scientific) equipped with an EASY-Spray™ electrospray source (held at constant 35ºC). Chromatography of peptides prior to mass spectral analysis was accomplished using capillary emitter column (PepMap® C18, 3µM, 100Å, 150x0.075mm, Thermo Fisher Scientific) onto which two microliters of extracted peptides was automatically loaded. NanoHPLC system delivered solvents A: 0.1% (v/v) formic acid , and B: 99.9% (v/v) acetonitrile, 0.1% (v/v) formic acid at 0.50 µl/min to load the peptides (over a 30 minute period) and 0.3 µl/min to elute peptides directly into the nano-electrospray with gradual gradient from 0% (v/v) B to 30% (v/v) B over 80 minutes and concluded with a five minute fast gradient from 30% (v/v) B to 50% (v/v) B at which time a four minute flash-out from 50-95% (v/v) B took place. Total run time of 150 minutes encompassed column conditioning at 95% B for one minute and equilibration at 100% A for 30 minutes. As peptides eluted from the HPLC-column/electrospray source survey MS scans were acquired in the Orbitrap with a resolution of 120,000 followed by CID-type MS/MS with 2.0 AMU isolation and 10 millisecond activation time with 35% normalized collision energy fragmentation of 30 most intense peptides detected in the MS1 scan from 350 to 1800 m/z; redundancy was limited by dynamic exclusion. Monoisotopic precursor selection and charge state screening were enabled and +1 and undefined charge states were rejected.

Data analysis and cross-linking assignment

Raw MS/MS data were converted to mgf file format using MSConvert (ProteoWizard: Open Source Software for Rapid Proteomics Tools Development) for downstream analysis. Resulting mgf files were used to search against user defined *C. atrox* venom proteome or liver proteome (translated transcriptome) amino acid sequence database with a list of common contaminants (172 total entries) using in-house *Mascot* search engine 2.7.0 (Matrix Science) with variable Methionine oxidation, Asparagine and Glutamine deamidation plus fixed cysteine Carbamidomethylation. Peptide mass tolerance was set at 10 ppm and fragment mass at 0.8 Da. Protein annotations, significance of identification, and spectral based quantification was done with help of Scaffold software (version 4.11.0, Proteome Software Inc., Portland, OR). Peptide identifications were accepted if they could be established at greater than 97.0% probability to achieve a False Discovery Rate (FDR) less than 1.0% by the Scaffold Local FDR algorithm. Protein identifications were accepted if they could be established at greater than 61.0% probability to achieve an FDR less than 1.0% and contained at least two identified peptides. Protein probabilities were assigned by the Protein Prophet algorithm (11). Proteins that contained similar peptides and could not be differentiated based on MS/MS analysis alone were grouped to satisfy the principles of parsimony. Proteins sharing significant peptide evidence were grouped into clusters.

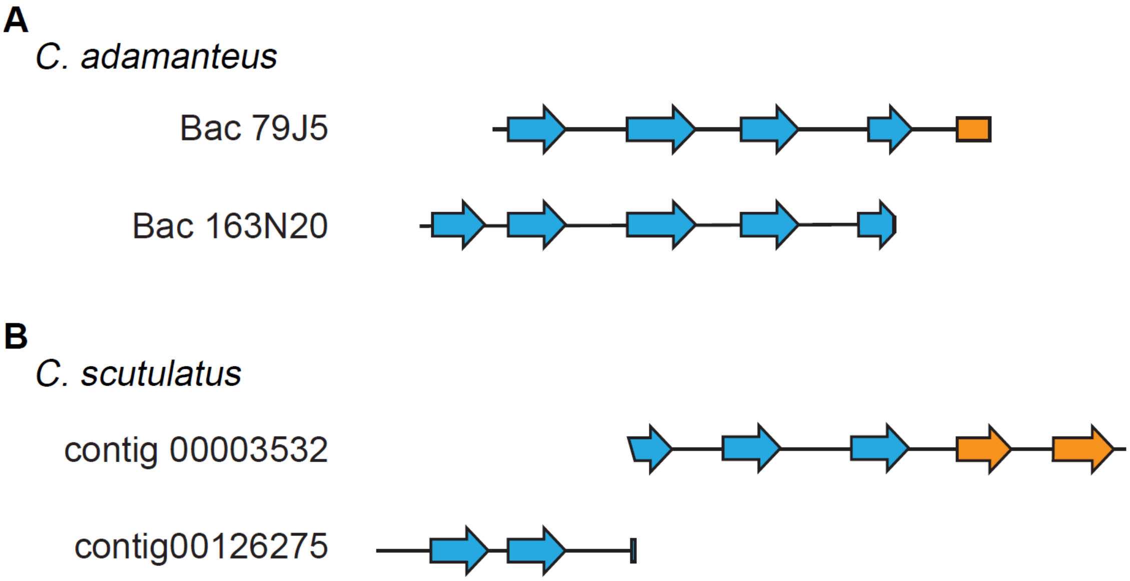

Fig. S1. Assembly of the fetua gene complex in Crotalus species. (A) Two overlapping BAC clones spanned the fetua locus in C. adamanteus to reveal five fetua genes (blue arrows). (B) The fetua locus in C. scutulatus was retrieved on two separate contigs from the C. scutulatus genome and shows at least five fetua genes and two fetub genes (orange arrows).

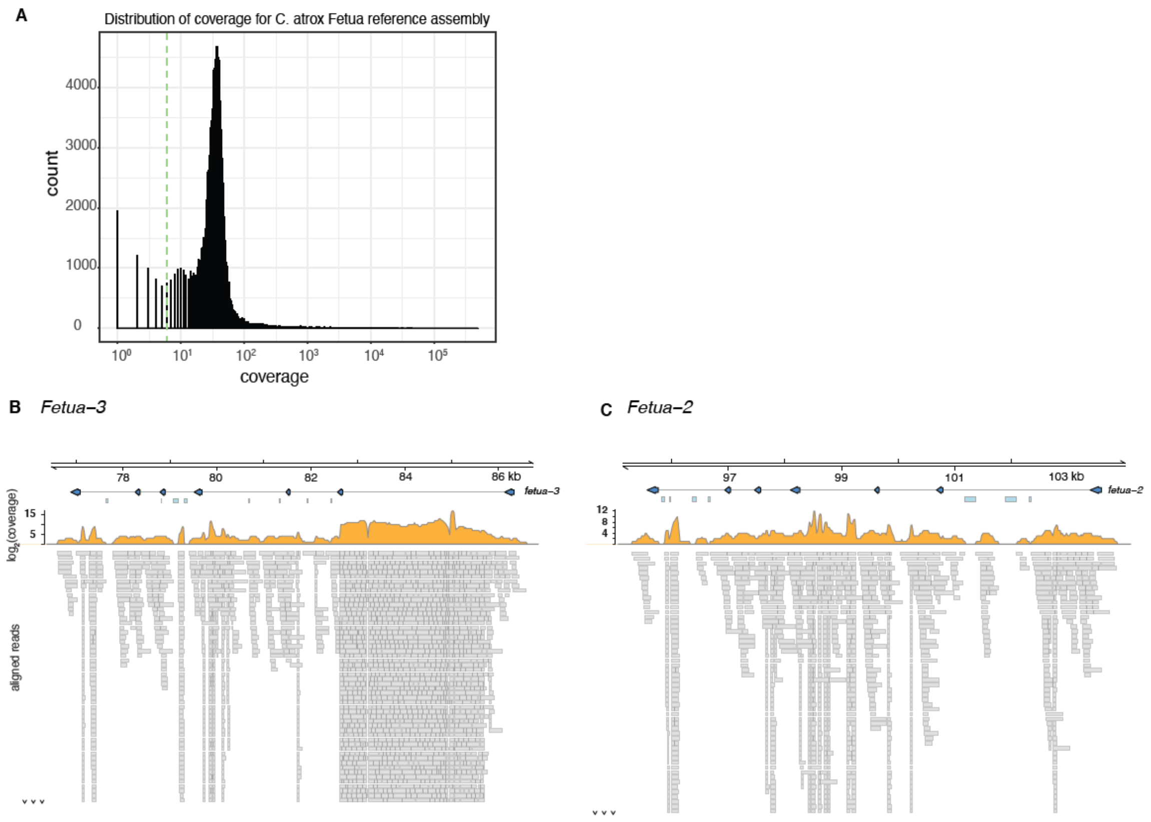

Fig. S2. Analysis of read coverage for reference guided assembly of *C. atrox Fetua* gene complex. (A) The distribution of coverage across the region. The mapped read coverage across the 220210 nucleotide (nt) region spans a wide range with a minimum of 0 (5046 nt) to a maximum of 485571 (mean = 11400, median = 46). The majority (54%) of nucleotides are covered by 10 to 100 reads but there are also regions with low or high coverage. The dashed green line shows the fifth percentile coverage level (6). (B-C) Log transformed read coverage (orange filled line plot) at the *fetua-3* (B) and *fetua-2* (C) loci with aligned reads shown as grey rectangles. Genes are shown as a grey line connecting exons (blue arrows) with masked zero coverage regions (light blue boxes) highlighted below the gene models.

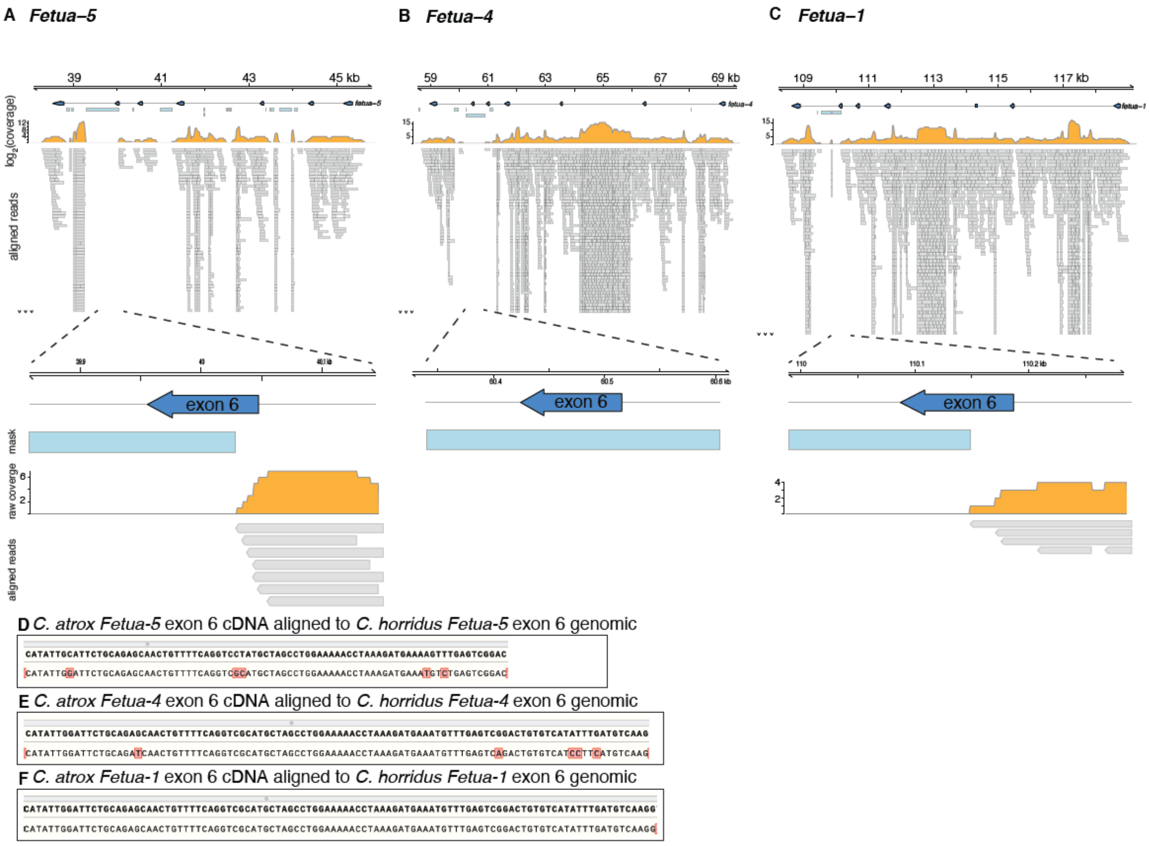

Fig. S3. (A - C) Log transformed read coverage (orange filled line plot) at the *fetua-5* (A), *fetua-4* (B) and *fetua-1* (C) genes with zoomed views (dashed line callouts) of exon 6 (blue arrow) and overlapping zero coverage masks (light blue rectangles). (D -F) Alignments of *C. atrox fetua-5* (D), *fetua-4* (E) and *fetua-1* (F) exon 6 cDNA sequences (bottom sequence with SNPs highlighted in pink boxes) to the corresponding *C. horridus* genomic sequence. The presence of these regions in the cDNA sequences supports the conclusion that exon 6 for these genes is present in the genome and their absence in our sequencing data is due to technical issues.

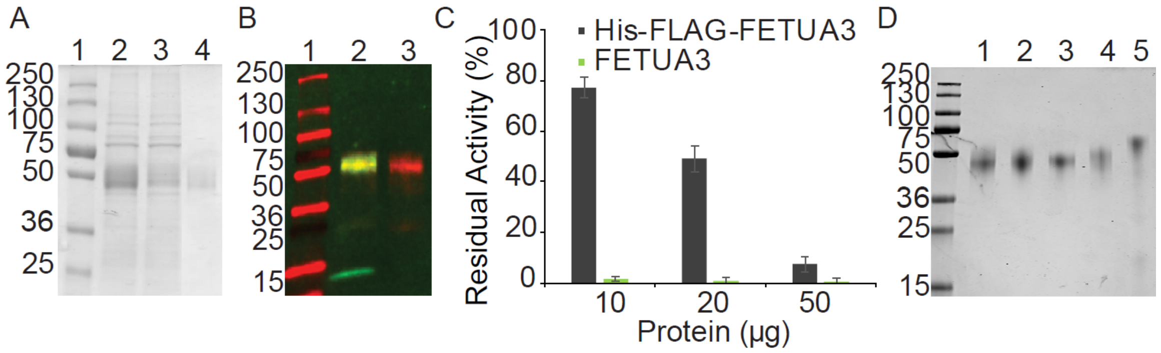

Fig. S4. Removal of the His-FLAG tag is necessary for optimal recombinant FETUA activity. (A) Coomassie stained SDS-PAGE of various fractions of recombinant *C. atrox* FETUA-3 purification. Lane 1 – protein standard, lane 2 – supernatant from recombinant FETUA-3 expressing cell line, lane 3 – flow-thorough from FLAG purification, lanes 4 – affinity purified His-FLAG-FETUA-3 protein. (B) Fluorescent Western blot of purified recombinant FETUA-3 blotted with anti-FLAG (green) and anti-FETUA-3 (red) antibodies. Lane 1 – protein standard, lane 2 – purified His-FLAG-FETUA-3 protein (yellow, detected by both antibodies), and lane 3 – enterokinase digested (untagged) FETUA-3 protein. (C) A comparison of the inhibitory activity of His-FLAG-FETUA-3 and FETUA-3 (tag removed) on *C. atrox* whole venom proteolytic activity on Azocoll substrate. (D) Coomassie stained SDS-PAGE of recombinant, tag-free *C. atrox* proteins, Lane 1 – FETUA-1, Lane 2 – FETUA-2, Lane 3 – FETUA-3, Lane 4 – FETUA-4, Lane 5 – FETUA-5.

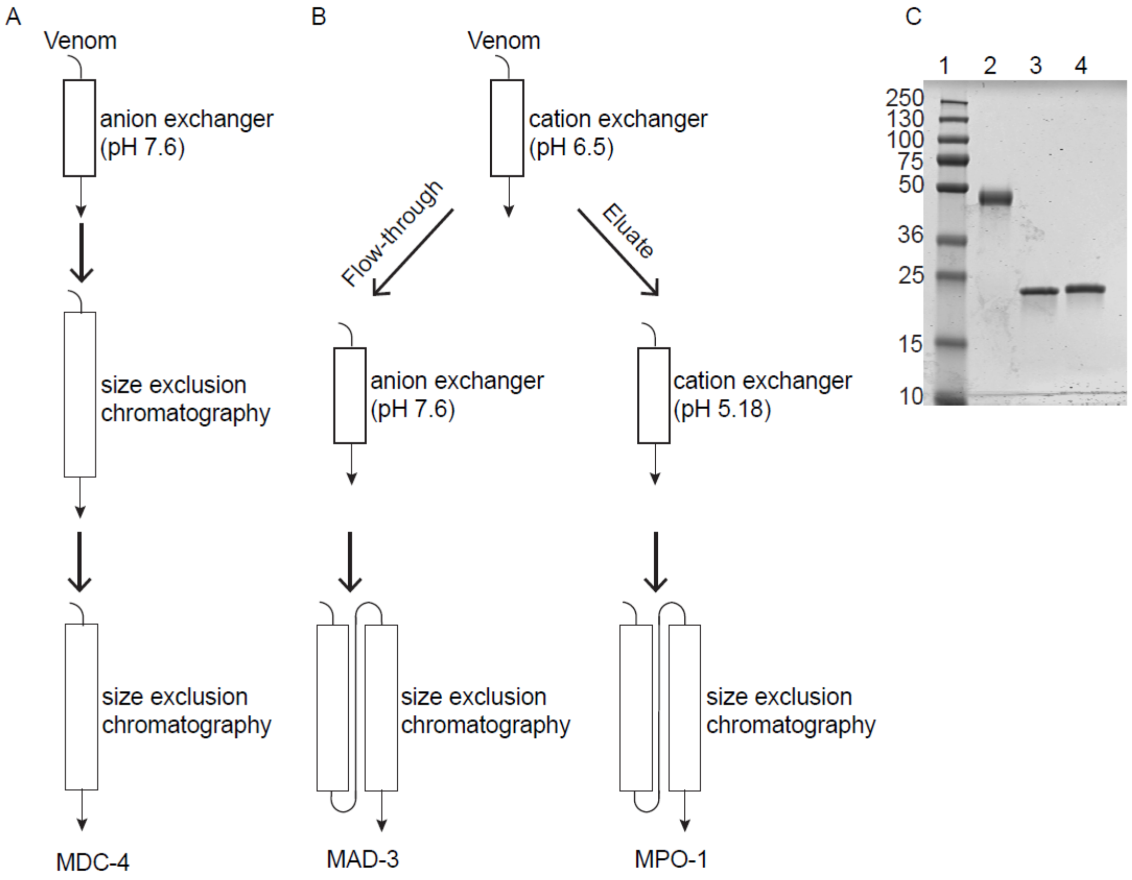

Fig. S5. Purification of SVMPs from *C. atrox* venom. (A) Schematic of MDC-4 purification from venom. (B) Schematic for purification of MAD-3 and MPO-1 from venom. (C) Purified SVMPs from venom analyzed by SDS-PAGE. Lane 1– Protein standard, lane 2 – MDC-4, lane 3 – MAD-3, lane 4 – MPO-1. See Supplementary Data 2 for Mass Spectrometry analyses of purified venom proteins.

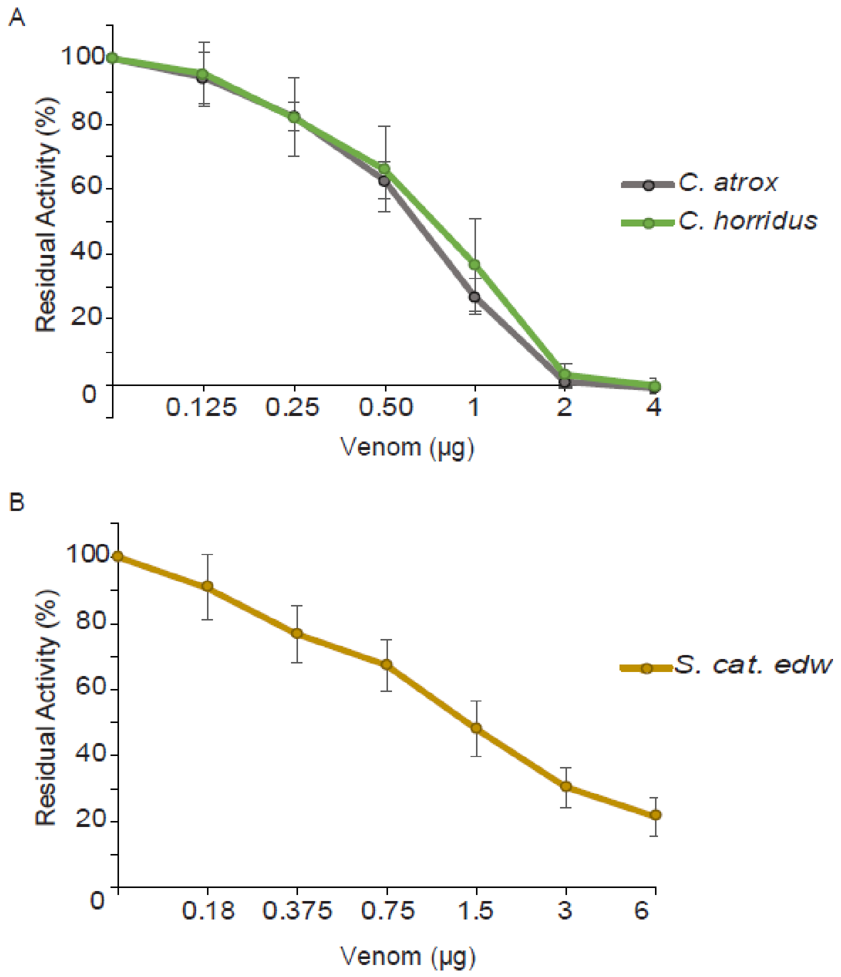

Fig. S6. *C. atrox* FETUA-3 shows cross-species inhibition of metalloproteinase activity. (A) Ten micrograms (µg) of whole *C. atrox* venom and fifteen micrograms (µg) of *C. horridus* venom were preincubated with FETUA-3 and was assayed for proteolysis of Azocoll substrate. (B) Twenty micrograms (µg) of *Sistrurus catenatus edwardsii* venom was assayed metalloproteinase activity on Azocoll in the presence of FETUA-3. The activity of each of the venoms were normalized to the activity observed with two microgram of *C. atrox* MDC-4. Error bars indicate standard deviation.

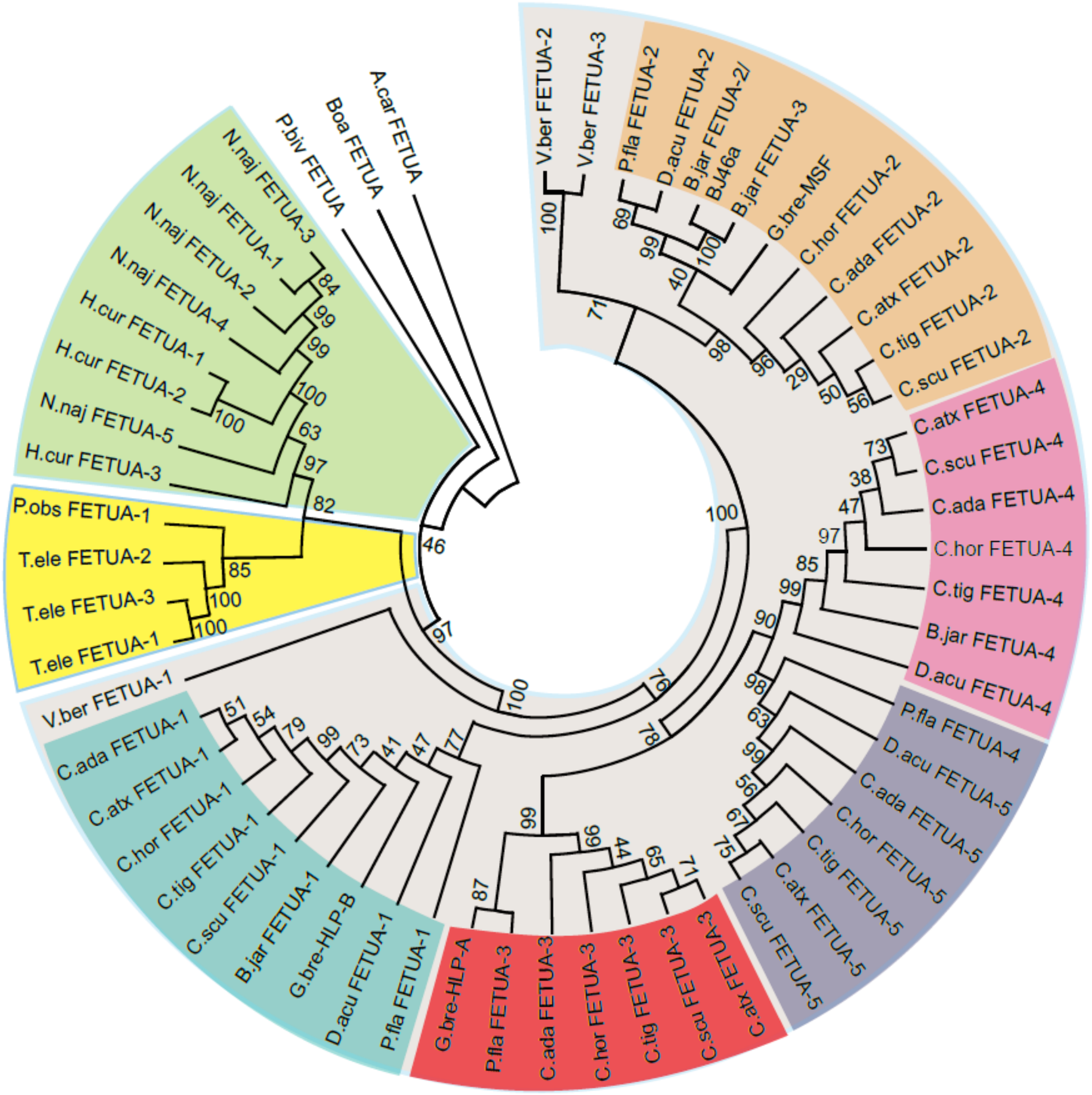

Fig. S7. *fetua-3* belongs to a different paralog group than other crotalid SVMP inhibitors. A DNA phylogeny of full-length FETUA proteins was constructed using General Time Reversible (GTR) model. Taxa are color-coded: Colubrids – yellow; Elapids – green; and Viperids – grey. Paralog genes in viperid species are marked with similar colored arcs: FETUA-1 – teal; FETUA-2 – orange; FETUA-3 – red, FETUA-4 – pink; and FETUA-5 – purple. Numbers at the nodes indicate the bootstrap values of 1000 replicates

Supplemental data

S. Data. 1: (A) List of primers used to recover cDNA clones from *C. atrox* liver mRNA (B) Primers used to screen BAC libraries for FETUA loci in *C. adamanteus* and *C. atrox*

A. cDNA -PCRs

Target	5' primer	3'primer	Amplicon
FETUA-5	ATGAATTCCCTGGTAGCTCTCGTG	oligo-dT	full length
FETUA-3	ATGAATTCCCTGGTAGCTCTCGTG	oligo-dT	full length
FETUA-1	ATGAATTCCCTGGTAGCTCTCGTG	oligo-dT	full length
FETUA-4a	GAGTCTTCCTTCTTTCCAACTAGTG	TAAAATACCGTCGTAAACCTCTCCG	TSS - 227bp
FETUA-4a	CCGGAGAGGTTTACGACGGT	TCTCAATGCTCTCTAGTATGGCTGG	202bp- 3'UTR
FETUA-2a	GAGTCTTCCTTCTTTCCAACTAGTG	TTTAAGGTAGACTGCCACCAAATC	TSS-160bp
FETUA-2a	TCTCAAGTGAGGGGGGATTTAGAATGT	oligo-dT	60bp-stop

B . BAC Library screen

Target	5' primer	3'primer	Amplicon
FETUA Ex 5-7	GTTTGCTATTGTGGAGGTTAACTGCACT	CACCACACATTCATCAGTACTGTTGC	~2Kb

S. Data. 2: Identification of the purified SVMP proteins from *C. atrox* venom via Mass Spectrometry by LC-MS/MS. Purified fraction (A) MDC-4, (B) MAD-3a/b, and (C) MPO-1(higher ~20kDa and lower ~13kDa bands). The highest number of peptides within each sample is highlighted in grey.

(A) MDC-4

Proteins	Exclusive Spectrum Count	Total Spectral Count	Total Unique Peptide Count
atx_mdc-4	1844	1995	49
atx_mdc-5a	3	55	9
atx_mdc-5b	4	56	11
atx_mdc-2	1	46	4
atx_mad-3a	3	11	9
atx_mad-3b	1	8	7
atx_mdc-8a	4	5	3
atx_mdc-8c	5	6	3
atx_mad-2b	1	4	4
atx_mpo-1	0	2	2
atx_mdc-3b	1	4	4
atx_mdc-3a	1	4	4
atx_gA1	15	15	6
atx_mdc-6a	4	13	8
OXLA_CROAT_Laao	14	14	10
VSP1_CROAT_svsp_catroxase-I	16	16	7
atx_mdc-6d	9	10	6
atx_mad-5b	6	8	6
VSPA1_CROAT_Alpha-fibrinogenase-A1	9	9	1
VSP2_CROAT_svsp_catroxase-2	7	7	6
VSPC1_CROAT_svsp_catroxbin-1	2	2	1
TXVE_CROAT_sVEGF_Cratrin	1	1	1
atx_mdc-6e	1	1	1

(B) MAD-3

Protein	Exclusive Spectrum Count	Total Spectral Count	Total Unique Peptide Count
atx_mpo-1	26	29	13
atx_mad-3a	66	248	11
atx_mad-3b	48	230	12
atx_mad-6	12	12	2

(C) MPO-1

Protein	Exclusive Spectrum Count	Total Spectral Count	Total Unique Peptide Count
atx_mpo-1	501	613	25
atx_mad-5a	1	1	1

S. Data. 3(A): Assignment of the FETUA-3 Affinity purified total eluate fractions of *C. atrox* Venom to SVMP genes via Mass Spectrometry by LC-MS/MS

Protein name	Protein identification probability	Peptide sequence	Number of Spectra
atx_mad-1b	97.90%	VTTGSQCAEGLCCDQCK	1
atx_mad-3a	100.00%	RHDNAQLLTSIDFDGPTIGLAYIGSICDPK	3
	100.00%	RHDNAQLLTSIDFDGPTIGLAYIGSICDPKR	1
	100.00%	RSTGVVQDHSEINLR	19
	100.00%	STGVVQDHSEINLR	15
	100.00%	YFSDCSYIQCWNFIMNQKPQCILK	6
	100.00%	YVELFIVVDHGMFTK	25
atx_mad-3b	100.00%	DGICDPKR	7
	100.00%	ISHDNAQLLTSTDFNR	6
	100.00%	QAIGLAYR	3
	100.00%	QAIGLAYRDGICDPK	1
	100.00%	RSTGVVQDHSK	2
	100.00%	STGVVQDHSK	6
	100.00%	VSMVDRNDDTCTGQSADCPR	2
	100.00%	YFSDCSYIQCWDFIMNQNPQCILK	15
	100.00%	YVELFIVVDHGMYTK	43
atx_mad-4a	100.00%	AWVYEIFNTINEIFQR	1
	100.00%	SHDNAQLLTSIDLDGPTIGLAYIGGICDPK	3
atx_mad-4b	100.00%	AWVYEIVNTINEIFQR	2

	100.00%	IIVQSSADITLDLFGTWR	4
	100.00%	NLCCDAATCK	1
	100.00%	SHDNAQLLTSINFDGQTIGWAYIGGICDSK	35
	100.00%	SHDNAQLLTSINFDGQTIGWAYIGGICDSKR	17
	100.00%	STGVVQDFSPINFLVAITMAHEMGHNLGMTHDENYCSCGGFACIMSPVISPQPSK	0
atx_mad-5a	100.00%	AYTGSMCDPR	4
	100.00%	AYTGSMCDPRK	1
	100.00%	ISHDNAQLLTAINFQENIIGR	21
	100.00%	ISHDNAQLLTAINFQENIIGRAYTGSMCDPR	1
	100.00%	IWVHEIVNTINVFYR	6
	100.00%	YIELVIVADHR	29
	100.00%	YNSNLNTIR	9
	100.00%	YNSNLNTIRIWVHEIVNTINVFYR	1
atx_mdc-2	100.00%	AAKDECDMADVCTGR	1
	100.00%	ACSNGQCVDVTTPY	2
	100.00%	CPIMADQCIALFGPGATVSQDACFQFNR	4
	100.00%	DECDMADVCTGR	2
	100.00%	DHREFLIK	1
	100.00%	DTCTCGTRPCVMAGALSCEASFLFSDCSQK	1
	100.00%	EGNHYGYCR	1
	100.00%	IACEPQDVK	1
	100.00%	LRQGAQCAEGLCCDQCR	1
	100.00%	MYDIVNVITPIYHR	1

	100.00%	NMPQCILK	1
	100.00%	NNNGYCYNGK	2
	100.00%	NPCNIYYSPNDEDK	1
	100.00%	QGAQCAEGLCCDQCR	2
	100.00%	SHDNAQLLTGINFNGPTAGLGYLGGICNTMYSAGIVQDHSK	5
	100.00%	TCRDPCCDATTCK	2
	100.00%	TDVVSPAVCGNYFVEVGEECDCGSPR	1
atx_mdc-3a	99.20%	LHSWVECESGECCDQCR	1
atx_mdc-4	100.00%	AMVTKNNGDLDK	2
	100.00%	ASMSECDPAEHCTGQSSECPADVFHK	35
	100.00%	DNSPGQNNPCK	45
	100.00%	ENGNKIPCAPEDVK	14
	100.00%	FVELFLVVDK	35
	100.00%	GNYYGYCR	10
	100.00%	GNYYGYCRK	12
	100.00%	HDNAQLLTAIDLDR	55
	100.00%	HDNAQLLTAIDLDRVIGLAYVGSMCHPK	11
	100.00%	HDNAQLLTAIDLDRVIGLAYVGSMCHPKR	0
	100.00%	ITVKPEAGYTLNAFGEWR	101
	100.00%	ITVKPEAGYTLNAFGEWRK	21
	100.00%	ITVKPEAGYTLNAFGEWRKTDLLTR	1
	100.00%	KENGNKIPCAPEDVK	4
	100.00%	KHDNAQLLTAIDLDR	75

	100.00%	KHDNAQLLTAIDLDRVIGLAYVGSMCHPK	8
	100.00%	KKHDNAQLLTAIDLDR	3
	100.00%	KTDLLTRK	5
	100.00%	LYCKDNSPGQNNPCK	1
	100.00%	MFYSNEDEHK	50
	100.00%	MYEIVNTVNEIYR	124
	100.00%	MYEIVNTVNEIYRYMYIHVALVGLEIWSNEDK	6
	100.00%	MYEIVNTVNEIYRYMYIHVALVGLEIWSNEDKITVKPEAGYTLNAFGEWR	1
	100.00%	NGQPCLDNYGYCYNGNCPIMYHQCYDLFGADVYEAEDSCFER	136
	100.00%	NQKGNYYGYCR	1
	100.00%	TRMYEIVNTVNEIYR	3
	100.00%	TRMYEIVNTVNEIYRYMYIHVALVGLEIWSNEDK	1
	100.00%	VCSNGHCVDVATAY	32
	100.00%	VIGLAYVGSMCHPK	91
	100.00%	VIGLAYVGSMCHPKR	1
	100.00%	YMYIHVALVGLEIWSNEDK	102
	100.00%	YMYIHVALVGLEIWSNEDKITVKPEAGYTLNAFGEWR	4
	100.00%	YNPFRFVELFLVVDKAMVTK	1
atx_mdc-5a	100.00%	AFDSNEDDHK	1
	100.00%	AHVASMCEPK	1
	100.00%	GDDDGYCR	4
	100.00%	GDDDGYCRK	2
	100.00%	HDNAQLLTAIDFSGSTIGK	1

	100.00%	RSECDIAESCTGQSDDCPTDDFHR	3
	100.00%	SECDIAESCTGQSDDCPTDDFHR	1
	100.00%	VCSNGHCVDVASAY	4
atx_mdc-5b	100.00%	AYIASMCDPK	5
	100.00%	FCTNGHCVDVATAY	2
	100.00%	GDDYGYCRK	1
	100.00%	LYCNDNSPGQNNPCK	1
	100.00%	RSECDIAESCTGQSADCPMDDFHK	2
atx_mdc-6a	100.00%	LFCNVNDFLCR	7
	100.00%	NRQCVDVTTAYK	1
	100.00%	QCVDVTTAYK	1
	100.00%	RYVELVIVADHR	12
	100.00%	YVELVIVADHR	1
atx_mdc-6b	100.00%	EQPSYEFSDCSQNQYLR	1
	100.00%	YSADPDYGMVNHGTK	1
atx_mdc-6c	100.00%	LFCEVNNFPCR	1
atx_mdc-6d	100.00%	ENGVNIPCSYEDVK	2
	100.00%	FALVGLEIWSNGDK	1
	100.00%	ITVQSSAYNTLDSFEEWRETDLLTR	2
	100.00%	LFCEFNNFPCQYK	2
	100.00%	RHDNAQLLTAIDFDGR	2
	100.00%	SECDIAESCTGQSADCPTDDFHR	3
	100.00%	TLGLASVSSMCNQK	1

	100.00%	YSEDLDYGMVDHGTK	2
atx_mdc-6e	99.90%	DDCDMAESCTGR	1
atx_mdc-7	100.00%	DDCDMADLCTGQSAECPTDR	4
	100.00%	DEDIGMVLPGTK	2
	100.00%	GSSYGYCR	1
	100.00%	GSSYGYCRK	1
	100.00%	GSVGVIQEHSTINLLMAVTMAHEMGHNLGMDHDIK	2
	100.00%	IPCPPQDVK	1
	100.00%	IYEIVNTMNEIYIPLNIHVALVR	10
	100.00%	KYVEFVVVLDHGMYTK	4
	100.00%	LFCFPNKPGEK	2
	100.00%	LTPGSQCADGVCCDQCR	4
	100.00%	NGHPCLNNK	9
	100.00%	NQCISFFGPSATVAK	3
	100.00%	NVCNVIYMSR	3
	100.00%	NVCNVIYMSRDEDIGMVLPGTK	5
	100.00%	RPQCILNEPLR	2
	100.00%	RPQCILNEPLRTDIVSPPVCGNELLEVGEECDCGSPGNCQNPCCNATTCK	2
	100.00%	SHDNAQLLTTIDFDGDTVGLAYVR	8
	100.00%	VCNSNGQCVDVNR	10
	100.00%	VCNSNGQCVDVNRAY	2
	100.00%	YCTCGAPSCIMADTLSHQPSK	3
	100.00%	YVEFVVVLDHGMYTK	7

atx_mdc-8b	100.00%	SECDIAESCTGR	1
atx_mdc-8c	100.00%	CPTLDHQCISFFGSNAAVAPDVCFDFNLK	1
	100.00%	FSDCSRDEHWR	2
	100.00%	GEGNFYCR	1
	100.00%	HDNAQLLTAIDLDGPTIGLAR	1
	100.00%	IFPCAPQDK	1
	100.00%	IFPCAPQDKK	2
	100.00%	LFCVLGPTGNTISCQSTSSQSDLDIGMVDLGTK	3
	100.00%	LRPGTQCEDGECCEQCQFR	1
	100.00%	NGQPCLNNNGYCYNGK	8
	100.00%	RHDNAQLLTAIDLDGPTIGLAR	1
	100.00%	RHDNAQLLTAIDLDGPTIGLARVGSMCDPK	0
	100.00%	RSTGIVQDHSK	1
	100.00%	SECDIAESCTGQSADCPTDNFQR	3
	100.00%	STGIVQDHSK	4
atx_mpf-1a	100.00%	LIFELGYR	1
	100.00%	LKPGTQCEDGVCCDR	1
atx_mpo-1	100.00%	AYTSSMCNPR	29
	100.00%	DHSPINLLVGVTMAHELGHNLGMNHDGDK	51
	100.00%	GASLCIMRPGLTPGR	50
	100.00%	ITTNPSVEDHCYYR	2
	100.00%	KRVHELVNTINGFYR	3
	100.00%	KSHDHAQLLTAINFEGK	2

	100.00%	NTLNSFGEWR	27
	100.00%	NTLNSFGEWREADLLR	2
	100.00%	RVHELVNTINGFYR	17
	100.00%	SHDHAQLLTAINFEGK	112
	100.00%	SHDHAQLLTAINFEGKIIGR	1
	100.00%	SHDHAQLLTAINFEGKIIGRAYTSSMCNPR	3
	100.00%	SLNIDVSLTDLEIWSDQDFITVQSSAK	50
	100.00%	SVGIVKDHSPINLLVGVTMAHELGHNLGMNHDGDK	0
	100.00%	VFMKYNSDLNIIR	1
	100.00%	VHELVNTINGFYR	45
	100.00%	YIELVVVADHRVFMK	3
	100.00%	YNSDLNIIR	20
	100.00%	YNSDLNIIRK	10

S. Data. 3(B): Assignment of the FETUA-3 Affinity purified fraction (gel excised, upper band) of *C. atrox* Venom to SVMP genes via Mass Spectrometry by LC-MS/MS

Protein name	Protein identification probability	SL #	Peptide sequence	Number of Spectra
atx_mad-3b	98%		QAIGLAYR	1
atx_mad-5a	99%		YNSNLNTIR	1
atx_mdc-2	99%		EGNHYGYCR	1
atx_mdc-3a	98%		VTLDSFGNWR	1
atx_mdc-4	100%		AMVTKNNGDLDK	3

	100%		AMVTKNNGDLDKIK	2
	100%		ASMSECDPAEHCTGQSSECPADVFHK	11
	100%		CADGKVCSNGHCVDVATAY	4
	100%		DNSPGQNNPCK	38
	100%		DNSPGQNNPCKMFYSNEDEHK	1
	100%		ENGNKIPCAPEDVK	7
	100%		ENGNKIPCAPEDVKCGR	3
	100%		FSKSGTECR	3
	100%		FVELFLVVDK	2
	100%		FVELFLVVDKAMVTK	1
	100%		GNYYGYCR	23
	100%		GNYYGYCRK	4
	100%		HDNAQLLTAIDLDR	8
	100%		HDNAQLLTAIDLDRVIGLAYVGSMCHPK	6
	100%		HDNAQLLTAIDLDRVIGLAYVGSMCHPKR	0
	100%		ITVKPEAGYTLNAFGEWR	9
	100%		ITVKPEAGYTLNAFGEWRK	3
	100%		KENGNKIPCAPEDVK	21
	100%		KHDNAQLLTAIDLDR	14
	100%		KHDNAQLLTAIDLDRVIGLAYVGSMCHPK	2
	100%		KKHDNAQLLTAIDLDR	35
	100%		KTDLLTRK	2
	100%		LKSGSQCGHGDCCEQCKFSK	1
	100%		LYCKDNSPGQNNPCK	14
	100%		MFYSNEDEHK	9
	100%		MFYSNEDEHKGMVLPGTK	15
	100%		MYEIVNTVNEIYR	31
	100%		NGQPCLDNYGYCYNGNCPIMYHQCYDLFGADVYEAEDSCFER	2

	100%		NQKGNYYGYCR	8
	100%		SGSQCGHGDCCEQCKFSK	2
	100%		SGSQCGHGDCCEQCKFSKSGTECR	1
	100%		SGTECRASMSECDPAEHCTGQSSECPADVFHK	0
	100%		TRMYEIVNTVNEIYR	4
	100%		VCSNGHCVDVATAY	7
	100%		VIGLAYVGSMCHPK	13
	100%		VIGLAYVGSMCHPKR	2
	100%		YMYIHVALVGLEIWSNEDK	3
atx_mdc-5a	100%		NQKGDDDGYCR	2
	100%		NQKGDDDGYCRK	1
	100%		RSECDIAESCTGQSDDCPTDDFHR	2
	100%		SECDIAESCTGQSDDCPTDDFHR	1
	100%		VCSNGHCVDVASAY	1
atx_mdc-5b	100%		FCTNGHCVDVATAY	1
atx_mdc-6a	100%		LFCNVNDFLCR	1
	100%		QCVDVTTAYK	1
atx_mdc-6d	100%		ENGVNIPCSYEDVK	1
	100%		KRHDNAQLLTAIDFDGR	1
	100%		SECDIAESCTGQSADCPTDDFHR	1
	100%		TLGLASVSSMCNQK	1
	100%		YSEDLDYGMVDHGTK	1
atx_mdc-7	100%		GKVCNSNGQCVDVNR	1
	100%		LFCFPNKPGEK	1
	100%		LTPGSQCADGVCCDQCR	1
	100%		NQCISFFGPSATVAK	1
	100%		RPQCILNEPLR	1
	100%		VCNSNGQCVDVNR	1

	100%		YKDDLDKIK	1
atx_mdc-8c	100%		IFPCAPQDKK	2
	100%		NGQPCLNNNGYCYNGK	8
	100%		RSTGIVQDHSK	1
	100%		SECDIAESCTGQSADCPTDNFQR	1
	100%		STGIVQDHSK	4
atx_mpf-1a	98%		ASQLNFTPEQR	1
atx_mpo-1	100%		GASLCIMRPGLTPGR	1
	100%		NTLNSFGEWR	1
	100%		SLNIDVSLTDLEIWSDQDFITVQSSAK	5
	100%		VHELVNTINGFYR	2
	100%		YNSDLNIIRK	1

S. Data. 3(C): Assignment of the FETUA-3 Affinity purified fraction (gel excised, lower band) of *C. atrox* Venom to SVMP genes via Mass Spectrometry by LC-MS/MS

Protein name	Protein identification probability	SL #	Peptide sequence	Number of Spectra
atx_mad-3a	100.00%		RSTGVVQDHSEINLR	2
	100.00%		STGVVQDHSEINLR	2
atx_mad-3b	100.00%		DGICDPKR	7
	100.00%		ISHDNAQLLTSTDFNR	4
	100.00%		QAIGLAYRDGICDPK	1
	100.00%		RSTGVVQDHSK	1
	100.00%		STGVVQDHSK	2
	100.00%		YFSDCSYIQCWDFIMNQNPQCILK	1

	100.00%		YVELFIVVDHGMYTK	2
atx_mad-5a	100.00%		AYTGSMCDPR	1
	100.00%		AYTGSMCDPRK	2
	100.00%		ISHDNAQLLTAINFQENIIGR	3
	100.00%		YIELVIVADHR	2
	100.00%		YNSNLNTIR	1
atx_mdc-4	100.00%		ASMSECDPAEHCTGQSSECPADVFHK	3
	100.00%		DNSPGQNNPCK	5
	100.00%		ENGNKIPCAPEDVK	1
	100.00%		FVELFLVVDK	1
	100.00%		GNYYGYCR	1
	100.00%		HDNAQLLTAIDLDR	1
	100.00%		ITVKPEAGYTLNAFGEWR	1
	100.00%		KENGNKIPCAPEDVK	3
	100.00%		KHDNAQLLTAIDLDR	1
	100.00%		KKHDNAQLLTAIDLDR	1
	100.00%		LYCKDNSPGQNNPCK	2
	100.00%		MYEIVNTVNEIYR	1
	100.00%		VCSNGHCVDVATAY	3
	100.00%		VIGLAYVGSMCHPK	2
	100.00%		YMYIHVALVGLEIWSNEDK	1
atx_mdc-6a	98.20%		YVELVIVADHR	1
atx_mpo-1	100.00%		AYTSSMCNPR	9
	100.00%		AYTSSMCNPRK	1
	100.00%		DHSPINLLVGVTMAHELGHNLGMNHDGDK	17
	100.00%		GASLCIMRPGLTPGR	30
	100.00%		KRVHELVNTINGFYR	3
	100.00%		KSHDHAQLLTAINFEGK	7

	100.00%		KSVGIVKDHSPINLLVGVTMAHELGHNLGMNHDGDK	0
	100.00%		NTLNSFGEWR	12
	100.00%		RKSHDHAQLLTAINFEGK	3
	100.00%		RVHELVNTINGFYR	4
	100.00%		SHDHAQLLTAINFEGK	12
	100.00%		SLNIDVSLTDLEIWSDQDFITVQSSAK	1
	100.00%		SVGIVKDHSPINLLVGVTMAHELGHNLGMNHDGDK	3
	100.00%		SYEFSDDSMGYYQSFLNQYKPQCILNKPLR	1
	100.00%		VFMKYNSDLNIIR	6
	100.00%		VHELVNTINGFYR	13
	100.00%		YNSDLNIIR	13
	100.00%		YNSDLNIIRK	1

S. Data. 4: (A) FASTA sequence of FETUA sequences used to construct phylogeny in snakes.

Protein	Species	Source	Accession	Sequence
A.car_FETUA	*Anolis carolinensis*	cDNA	XP_003225747.1	MKSLIALVLLGQILASKATSEPFPPLVSSLPDCDDPESEAAANFAV<br>DHINSHSLHGYKYTLSRIENVKVLPRRPTGKIYLLELDLAETKCHV<br>LSPTPIQNCSVRAKVEHAVEGDCDVKMLHLDGQYKVLSTKCHSS<br>PDSREDFEKVCPDCAPLALLDDVNVVNAVNTALAQYNTYNSTDH<br>HFELLEIARGRNSHMPPGTFVEFAIAATNCTEQEAKEHKDCHVM<br>TGEHAQFGFCKATIFKKPSGEGPALFAIFPNVVICDIFDKQVGHSH<br>THLTKHHLGKKIPSPGAGYSVLDLIHSHNNTLASHESHSAEVPVV<br>QASPVVKRAAEVPPVIQPALPHLQQCPGKYRHFDI*
B.con_FETUA	*Boa constrictor*	cDNA	http://dx.doi.org/10.5524/100060	MNSLIALVLLGQILGCTLSHHLGLSLDCNSKEAEQYAEQAVHHIN<br>AHNLHGYKQILNIIEDLNIVHRRPRGEVIFIEVNLLETKCHVLDPTPI<br>ENCTVRTQQEHAVETDCDVKLLSDEGITKVVAAKCHSSADSVED<br>PMKNCAHCSVLMPLNDPHVIEVVEYVLHKHNEQNPGNVYSVLEI<br>SRGQHRHEPESFYVEFAIVETNCSGQDAAEGHNGCHAKAAADA<br>HVGFCRATVFRKHTATEKLTDEKFESDCVIFEPKAGESHTHLIEH<br>HFGKNIPSPGHNHTILDLIHSHNDTNASHESHSSEALPEVAAPVV<br>KREVPETVIPPINQPTLPVKLCPGKVHHFKV*
P.biv_FETUA	*Python bivitattus*	cDNA	XP_007434930.1	MNSLIALVLLGQILGCTLSHHLLPQSDCNSEEAEHYAEEGVHYIN<br>THNLHGYKQTLNIIKDLHVLPRRPHGKVIFTELNLLETRCHVLDPT<br>PVENCTVRTQEEHAVEADCDVKLLSDEGVTKVVAVKCHSSPDS<br>VEDVKKLGPNYPILLPLNDPRVVEVVEYVLHKHNEQQPDHVFEV<br>LEITRGQHKHEPESYYVEFVFVETNCSEQESHDAHHDCHPKAAA<br>DAHVGFCKATVFRAHDATGKLTDEKFESDCVLFDQKVGETHVHL<br>IEHHLGKNIPSPGHGHTVLDLAHSHNDTNASHESHSAEVLPEVAA<br>PVVKRDVSPTLPPDDHLTPPVKLCPGKVHHFKV*
P.obs_FETUA	*Pantherophis obsoletus*	genomic DNA	WJSR01000052.1	MNSLVALVLLGQIIGCTFSHHLLPQLDCNSEEAERLAKLAVDYINE<br>HTLHGYRQALNIIKDAHVVPRRPSGKIIFLELSLLETVCHVLDPSPL<br>EKCTVRPQKYHAVEGDCDVKILSDEGIEKVVAVKCHSEPDSAED<br>VLFNCPICPILLPRNDPHVVESVEYVLHKHNEQLTGHSYEVLEISR<br>GQHKLEPEAYYVEFAIVETNCSAKEAHDNHHDCHPKAAGEGHL<br>GFCRATVFRSQAATEKPKDEKYESDCIIFDVKVGIPHAHLIEHHYA<br>KNIVSPGHNNTVLDLVHSHNHTSASHETHSHEHAATVAAPVVKR<br>EAPTEGPHDHTHATNLCPGKVHHFKI*

T.ele_FETUA-1	*Thamnophis elegans*	genomic DNA	GCA_009769535.1	MNSLVALVLLGQIIGCTLSHHLVPQFDCNGEEAEKLGNLAVSYIN<br>EHSLHGYKHVVNQIKDAHVLPRRPHGKVIFLELDLLETVCHVLDP<br>TPIENCSVRAQQYHAVEADCNVKLVSDEGVDKVVAAKCHSEPD<br>SVEDVRQHCPKCPILLSLSDPHVINSANYVLHKHNDKLPHHAYEV<br>LEISRGQQKFDPEGYYVEFAIVETNCTHQEAHDDNYHCDPKALG<br>EAHFGLCRATVFRSHAATGKPTDEQYESDCVIFDVKDGHSHSHL<br>VEHHFRKNIVSPGYNNTVLSLIHSHNHTSASHETHSHEHGTAIPP<br>HVAKREVPTVPSHDHTHPIKLCPGKVHHFKV*
T.ele_FETUA-2	*Thamnophis elegans*	genomic DNA	GCA_009769535.1	MNSLVALVLLGQIIGCTLSQHLEGWQFDCHDEEVEKLARVAVSYI<br>NEHSKHGYKQVVNQIKDAYALPQRPHGKINHLELNLLETVCHVL<br>DPTPAENCSVRAQNHHAVEADCHVKLVSDEGVDKVVAAKCHSQ<br>PDSVEDVRQHCPKCQILLSLSDPNVIDSANYVLHKHNDKLTHHAY<br>EILEISRGQQKFDPEGYYVEFPIVETNCTHQEAHDDTHHCHPKAL<br>GEAHFGFCRATVFRSHAATGDLTDEQYESDCVIFNVKDGHSHSH<br>LVEHHFRKSIVSPGYNNTVLDLVHSHNHTSASHETNSDEYGTAIP<br>PHAGKREVPTVPSHGHALIKLFCPGEIHHFKV*
T.ele_FETUA-3	*Thamnophis elegans*	genomic DNA	GCA_009769535.1	MNSLVALVLLGQIIGCTLSHHFGPQFDCNGEEAERFASVAVSYIN<br>EHSIHGYKHALNIIKDVHVLPRRPHGKVIFLELDLLETVCHVLDPT<br>PIENCSVRAQQYHAVEADCDVKLLSDEGVDKVVAAKCHSEPDSV<br>EDVRQHCPKCPILLSLSDPHVVDSANYVLHKHNDKLPHHAYEVL<br>EISRGQQKFDPEGYYVEFAIVETNCTHQEAHDDNYHCDPKALGE<br>AHFGLCRATVFRSHAATGKPTDEKYESDCVIFDVKDGHSHSHLV<br>EHHFRKSIVSPGYNNTVLDLVHSHNHTSASHETHSHEHGTAIPPH<br>VAKREVPTVPSHDHTHPIKLCPGKVHHFKV*
N.naj_FETUA-5	*Naja naja*	genomic DNA	GCA_009733165.1	MNSLVTLVLLGQIIGCTFSHHLLPQLDCNGEEAENLAELAEQYINE<br>HNLHGYKHTLNVIKDVHVLPRRPHGKVIFLNLEFLETVCHVSDPT<br>PIENCTVRPDYYHAVEAECDVRLLSDEDVVKVVASKCHSEADSV<br>ENVRQKCPKCPILIPLNDPHVVQSVEYVLRKYNEEHPEHVFEVLE<br>ISRGQHKYDPEAFYVEFAIVETNCSAQEAHDDHHDCHPKAAGEA<br>RAGFCRATVFRSHADLEKPKDELYESDCVIFDVKVGHSHTHLIEH<br>HFGKNIPSPGHNNTILDLVHSHNHTSASHESNSHEHVAATEVVAP<br>VAKREAPTAVSHDHTHATKLCPGRVHHFKV*

N.naj_FETUA-4	*Naja naja*	genomic DNA	GCA_009733165.1	MNSLVTLVLLGQIIGCTFSVSLLRKFDCNGEEAEELAKVAVKFINE HNLHGYKQTLNIIKEIDVRAPRPKPNVEKVVEIYLNLLETKCHVMD PTPVEKCTVRQQHEHAVEADCLVRIHRELGNKVTGAVCYSKPGS VLKVRQNCTKCPILIPLNDPHVVQSVEYVLRKHNEQLPNHAYEVL EISRGQHQYDPEAFYVEFAIVETNCSAQEAHDDHHDCHPKAAGE AHTGFCRATVFRSHAALEKPKDEKYESDCVIFDVKEGHSHSHLIE HHYGKNIASPGHNNTILDLVHSHNDTDTSNESDSLEAVLDPELLV KTEAPAEIAHDPTKAPPVCPGRVHHFEV*
N.naj_FETUA-3	*Naja naja*	genomic DNA	GCA_009733165.1	MNFLVTLVLLGQIIGCTFGTTLSQEFDCNGEEAEKLAKLAVKYIND HNLHGYKQTLNVIKEVDFPAPRPREIVEMVAEMTLNVLETKCHVL DPTPVENCTVRQQHEHAVEAECVVRIHRELGDKVSGANCHSKP GSVGNVRRNCTKCPILIPLNDPHVVQSVEYVLRKHNEQLPNHVY KVLEISRGQHQYDPEAFYVEFAIVETNCSAQEAHDDHHVCHPKA AGEGHTGFCRATVFRSHAALEKPKDEKYESDCVIFDVKKGHSHS HLIDHHYGKNIASPGQNNTVLDLVHSHNHKSSSHESLSVEVMAV PLPVVKREAPAEIAHAHAKAAPLCPGKVHHFKL*
N.naj_FETUA-2	*Naja naja*	genomic DNA	GCA_009733165.1	MNFLVTLVLLGQIIGCTFGTTLSQEFDCNGEEAEKLAKLAVKYIND HNLHGYKQTLNVIKEVDFPAPRPREIVEMVAEMTLNVLETKCHV MDPTPVENCTVRQQHEHAVEAECVVRIHRELGDKVSGANCHSK PGSVGNVRRNCMKCPILILLNDPHVLQSVEYVLRKHNEQLPNHV YKVLEISRGQHQYDPEAFYVEFAIVETNCSAQEAHDGHHVCHPK AAGEGRAGFCRTTVFRSHAALEKPKDEQYELDCVIFDDKKGHSH SHLIDHHYGKNIASPGHNNTVLDLVHSHNHKSSSHESLSLEAMTV PLPVVKREAPAEIAHAHANAAPLCPGKVHHFKL*
N.naj_FETUA-1	*Naja naja*	genomic DNA	GCA_009733165.1	MNFLVTLVLLGQIIGCTFGTTLSQEFDCNGEEAEKLAKLAVKYIND HNLHGYKQTLNVIKEVDFPAPRPREIVEMVAEMTLNVLETKCHVL DPTPVENCTVRQQHEHAVEAECVVRIHRELGDKVSGANCHSKP GSVGNVRRNCTKCPILIPLNDPHVVQSVEYVLRKHNEQLPNHVY KVLEISRGQHQYDPEAFYVEFAIVETNCSAQEAHDDHHVCHPKA AGEAHTGFCRATVFRSHAALEKPKDEKYESDCVIFDVKKGHSHS HLIDHHYGKNIASPGQNNTVLDLVHSHNHKSSSHESLSVEVMAV PLPVVKREAPAEIAHAHAKAAPLCPGKVHHFKL*

H.cur_FETUA-1	*Hydrophis curtus*	genomic DNA	GCA_019472885.1	MNSLVTLVLLCQIIGCMFSHHLSPQLDCNGEEAEKLAELAVHYIN<br>KHNLHGYKQALNVIKDVHVLPRRPSGKVIFLELDLLETECHVLDP<br>TPVENCTVRPQHYHAVKGDCDVKLLSDEGINKVVASKCHSDPDS<br>VEDVRQNCLNCPILIPLNDPHVVQSVEYVLHKHNEKQPSHAYEVL<br>EISRGQHKYEPEAFYVEFAIVETNCSAQEAHDDHHDCHPKAAGE<br>AHIGFCRATVFRSHATLEKPKDEQYESDCVIFDVKEGHSHTHLIE<br>HHFGRNIASPGENNTVLDLIHSHNHTSASHESHSHEHMVIPVVAA<br>PVAKREAPTEISHDHTHPPKLCPGKVHHFKV*
H.cur_FETUA-2	*Hydrophis curtus*	genomic DNA	GCA_019472885.1	MNSLVTLVLLAQIIECMFSVVLLKESDYNGEEAKKYGDLAVHYIN<br>QHNLHGYKQTLNVIKRVDFLPPPYVEKVVSVELDVLETECHVLDP<br>TPVENCTVRQQDHHAVKSECVVRIHRENGDKVFGANCHSKPDS<br>VENVRQNCSKSPILVPLNDPHVVQSVEYVLHKHNEKQPSHA*EV<br>LEISRGQHQYEPEAFYVEFAIVETNCSVQEAHDDHHDCHPKAAG<br>EAHIGFCRATVFRSHAALEKPKDEQYESDCVILDVKEGHSHTHLI<br>EHHYGKNIPSPGHNSVVLDLAHSHNHTSSSHESHSQERVVEVIV<br>LPLPVAKREAPTETAHDHAKAAPLCPGKVHHFKL*
H.cur_FETUA-3	*Hydrophis curtus*	genomic DNA	GCA_019472885.1	MNSLVTLVLLCQIIGCTLSLLLRKEFDCNGEEAKKFGDLAVHYINQ<br>HNLHGYKQTLNVIKRVDFLATRS*PYVEKVVSVELDVLETECHVL<br>DPTLSRIVPVRQQDHHAVKSECVVRIHRENGDKVFGANCHSKPD<br>SVENVRQNCSKSLILVPLNDPHVVQSVEYVLHKHNEKQPSHA*E<br>VLEISRGQHQDEPEAFYVEFAIVETNCSAQEAHDDHHDCHPKAA<br>GEAHIGFCRATVFRSHAALEKPKDEQYESDCVIFDVKEGHSHTH<br>LIEHHYGKNIPSPGHNSVVLDLAHSHNHTSSSHESHSQERVVEVI<br>VLPLPVAKREAPTETAHDHAKAAPLCPGKVHHFKL*
V.ber_FETUA-1	*Vipera berus*	genomic DNA	KN624939	MNSLIALLLLGQIIGCTFSHHLPSHGDCNGEDAKKWAHLAVHYIN<br>EHTLHGYKQDLNIIKDIHVLPRRPRGKIIFLELELLETVCHVLDPTP<br>LENCTVRPQHYHAVEGDCDVKIIHDEDVDKVVAAKCHSSPDSVE<br>DVRRNCPKCPILFPLNDPHVVDAIEYVLNKHNEKLSGHVYEVLEI<br>SRGQYTFEPEGFYVEFAIVESNCTAQEAQDDHHHCHPNRAGEE<br>HIGFCRATVVRSHASLEKLKDEQFESDCVIFDVKEGHSYSHLIEH<br>HIGKYSTSPGHNNTVLNLAHSHNHTSASHESHSHEHVVEVPVAV<br>AKREIPTNIPPHHTHPVNLCPGKVHHFKV*

V.ber_FETUA-2	*Vipera berus*	genomic DNA	KN624939	MNSLVALLLLSQIIGSTLSSQVRGDIDCNDKDAKDWAGKAVRYINEHKLHGYKQDLNVIKNIHILPWDGDLVAIYLKLNFLETECHVLDPTPVENCTVRPQHNHAVKIDCDVKIIYDIETHKDDVFVKCNSTPDSVENVQQNCPKCPILLPQNDPHVVDAVEYVLNKHNEKLSGHVYEVLEISRGQHTFEPEGFYVEFAIVESNCTAQEAQDDHHHCHPNTEGEDHIGFCRATVFRSHASLEKPKDEQFESDCVIFDVKKGHAHCYLIEQHVGKDSTSPGDNTNVLNLAHSHNHTRASHESHSDECVVGCPVAFVKKEVPTDISDHDTPSVKGCPGRVLHFNL*
V.ber_FETUA-3	*Vipera berus*	genomic DNA	KN624939	MNSLVALLLLGQVIGSTLSSQVRGDIDCNDKDAKHWADIAVRYINEHKLHGYKQDLNVIKNIHILTWDRDLVAGYLKLNLLETECHVLDPTPVEKCTVRPQHNHTVEMDCDVKIIYNVMTLKDEVSVKCNSTTDSVENVQRNCPKCPILLPRNDPHVVDAVEYVLNKHNEKLSGHVYEVLEISRGQHTFEPEGFYVEFAIVQSNCTAQEAQDDHHHCHPNTAGEDHIGFCRATVFRSHASLEKPKDEQFESDCVMFDVKEGHTHSYLIEQHVGKDSTSPGDNTTVLNLAHSHNHTRASHEFHSDECVVECPVAFVKKEVPTDISDHHTPPAKGCPGRVLHFKL*
D.acu_FETUA-1	*Deinagkistrodon actus*	genomic DNA	http://dx.doi.org/10.5524/100196	MNSLVALLLLGQIIGCTFSHHLTSQVDCNGEDAEKWGDMAVHYINEHNLHGYKQALNVIKEIHVLPRRPHGEIVFIEIEVLETQCHVLDETPVENCTVRPQHYHAVGGDCDVKIIHHEGVDKVVGAKCHSDPDSVEDVRRNCPNCPILLPLSDPRVVDCVEYVLSKHNEKLSGHIYEVLEISRGQHQYEPEAFYVEFVIVEVNCTAQEAHDDHHHCHPNTAGEDHIAFCRATVFRSLASLEKPKDEKFESDCVILNVKEGHAHSHLIQHHVGKYSISPGHNNTVLNLAHSHNHTSASHESHSHEHVAEVPVAVAKREVPTDIPHHHMHSHPVKLCPGKVHHFKL*
D.acu_FETUA-2	*Deinagkistrodon actus*	genomic DNA	http://dx.doi.org/10.5524/100196	MNSLVALLLLGQIIGSTLSSQVRGDLECDNKDAKEWADVAVRYINEHKLHGYKHALNVIKNIVVVPWDGDLVAVFLKLNLLDTECHVSDSTPVENCTVREQHNHAVEMDCDVKIMFDIETFKRDVFVKCHSTPDSVENVRRDCPKCLILLPPNDPRVVDSVEYVLSKHNEQLSGHIYEVLEILRGQYKYEPEAFYVEFVIVEVNCTAQEAHDDHHHCHPNTAGEDHIAFCRATVFRSHVGLEKPKDKKFESDCVILHVKDGHAHSHLIQQHIEKNSISPEHNITILNFIHPQNHTSTSHESHEHAAEVPVDFVKKELPTDISNRHTPPVKGCPGKVHHFEL*
D.acu_FETUA-4	*Deinagkistrodon actus*	genomic DNA	http://dx.doi.org/10.5524/100196	MNSLVALLLLGQIIGSTLSYLVKEEDLNCNSKAAKYRADQVVRYINDHKLHGYKQALNVIKIFHLLSPERFSTVFYLKLNLLDTECHVLDQTPVEKCTVRPQHNHAVEMDCDAKIIFNYDIYKDYIFVKCNSTP*YEPEAFYVEFVIVEVNCTAQEAHDDHHHCHPNTAGEDHIAFCRATVFRSHASLEKPKDEKFESDCVILNVKRGHAYSQLIQHHVEKNSISPG

				HNSTVLNLAHSHNHTRASHESHSDECVVECPVAFVKKNIQTNIPHHTHPVEGCPGRVFHFQL*
D.acu_FETUA-5	*Deinagkistrodon actus*	genomic DNA	http://dx.doi.org/10.5524/100196	MNSLVVLVLLGQVIGSTHSSMVKEEDLNCNSKAAKYRADQAVDYINEHNLHGYKQALNVIKIIRLLPSDGLSVIFNLKLNLLETKCHVLDPTPVENCTVRPQLNHAVETDCDVKIIYNIATFKDEFLVKCHSTPGSVENILRDCPKCPLLLPLSDPHVVDSVEYVLSKHNEKLSGHIYEVLEISRGQHQ*HIAFCRATVFRSHASLEKPKDEKFESDCVILHVKGGHAHSHLIQHHTGKYSTSPGHNSTILNVAHSHNYTSASHESHSHEHVAEVPVDSVKKEVPTDIPHHHTPPVDGCPGKVLHFKL*
P.fla_FETUA-1	*Protobothrops flavoviridis*	genomic DNA	BFFQ01000665	MNSLVALLLLGQIIGCTFSHHLPSHVPVDCNGEDAEKWADLAVDYINEHNLHGYKYAVNVINEIFVLPRRPHGKMILLELNLLETECHVLDQTPIKNCTVRPPHYHAVEGDCDVKIIHDEDVDKVVAAKCYSNPDSVEDVQQNCPKCPILLSLTDPHVVDSVEYVLEKHNAKLSGHIYEVLEISRGQHKYEPEAYYLEFVIVEVNCTAQEAHDDHHQCHPYTAGEEHIGFCRATVFRSHASLEKPKDEKFESDCVIFDIKEGHAHSHLIEHHVGKYSTSPGYNNTVLNLVHSHNHTSASHESHSHEHVAEVPVAVAKREVPTDIPHDHTHPVKLCPGKVHHFKL*
P.fla_FETUA-2/HSF	*Protobothrops flavoviridis*	genomic DNA	BFFQ01000665	MNSLVALVLLGQIIGSTLSSQVRGDLECDDKEAKNWADDAVRYINEHKLHGHKQALNVIKNICVVPWNGDLVAVFLELNLLETECHVLDPTPVEKCTVRQQHNHAVEMDCDAKIMFNVETFKRDVFVKCHSTPDSVENVRRNCSKCPILLPPNNPHVVDSVEYVLNKHNEKLSGHIYEVLEISRGQHKYEPEAYYLEFVIVEINCTAQEAHDDHHQCHPYTAGEDHIAFCRSTVFRSHASLEKPKDEKFESDCVILDVKDGHAHSHLIQQHIEKNSISPEHNITILNFVHPDNHTSTSHESHEHVAEVPVVFVKKELPTDISDHHTTPVKGCPGKVHHFEL*
P.fla_FETUA-3/HLP	*Protobothrops flavoviridis*	genomic DNA	BFFQ01000665	MNSLVALVLLGQIIGSTVSFQLGPNMDCNTKGTKDWADIGVHYINEHKLHGYKQALNVIKIFRLLPSDGRSVIFHFNLNLLETECHVLDSTPVENCTVRPQHNHAVEMDCNVRIIHDITTFEDEVFVKCSSTPGSVENILRDCPKCPILLSPNDPHVVDSVEYVLNKHNEKLSGHIYEVLEISRGQHKYEPEAYYLEFVIVEINCTAQEAHDDYHQCHPYTAGEDHIAFCRSTVFRSHASLEKPKDEKFESDCVILDVKEGHSHSHLIEHHVGKYSTSPGYNSTDECVVECPVAFVNKEVPTDISDHNTPPVKGCPGRVLHFQL*

P.fla_FETUA-5	*Protobothrops flavoviridis*	genomic DNA	BFFQ01000665	MNSLVALVLLGQIIGSTHSYMVKEEDLNCNSKGAKY*ADQAVRYI NEHNLHGYKQALNVIKIFRLLPSAGFSVIIYLKLNLLETECHVLDST PVENCTVRQQHNHAVETDCDVKIIYNIMTFKDEFLVKCHSTPGSV ENILRDCPKCPILLSLSDPHVVDSVEYVLEKHNAKLPGHIYEVFEI SRGQHKYEPEALYLEFVIVEVNCTAQEAHDDHHQCHPYTAGED HIAFCRSTVFRSHASLEKPKDEKFESDCVILDVKEGHAHSHLIQQ HIEKNSISPEHNITILNFVHPHNHTSTSHESHEHVADVPVAFVKKE LPTDISDHHTTPVKGCPGKVHHFEL*
G.blo_MSF	*Gloydius blomhoffi*	cDNA	Q5KQS4	MHFLVALVLLGQIIGSTLSSQVRGDLECNDREAKEWADQAVRYIN EHKLHEYKQALNVIKNIVVVPWNGDLVAVFLKLNLLETECHVLDP TPVEKCTIRPQQNHAVEMDCDAKIMFDVETFKQDVFVKCHSTPD SVEDVRRNCLKCPILLSPSDPHVVDSVEYVLNKHNEQLSGHVYE VLEISRGQHKYEPEAFYVEFAIVEVNCTAQEAHDDHHHCHPNTA GEDHIAFCKATVFRSHASLEKPKHENFESDCVILDVKEGHAHSHL IEHHIGKYSTSPGQNSTVECVAECPVAFVNKEVPTDISDRHTTPV KGCPGKILHFQL*
G.blo_HLP-B	*Gloydius blomhoffi*	cDNA	BAD88538	MNSLVALVLLGQMIGSTLSHHLQSHVDCNGEDAEKWADMAVHYI NEHNLHGYKQVFNVINEIHVLPRRPRGKIIILELKLLETECHVLDPT PVENCTVRPPHYHAVEGDCDVKILHDEGVDKVIGAKCHSDPDSV EDVRRNCPKCPILLPLSDPHVVDSVEYVLNKHNEKLSGHVYEVL EISRGQHKYEPEAFYVEFAIVEVNCTAQEAHDDHHHCHPNTAGE NHIGFCRATVFRSHASLEKPKDEQFESDCVIFDVKEGHAHSHLIE HHIGNYNTSPGHNNTVLNLAHSHNHTSASHESHSHEHVAEVPVA VAKREVPTNTPHDHTHPVKLCPGKVHHFKL*
G.blo_HLP-A	*Gloydius blomhoffi*	cDNA	BAD88537	MNSLVALVLLGQIIGSTLSFQLGPNMDCNTKGTKDWADIGVRYIN EHKLDGYKNALNIIKIFRLLPSDGRSVIVHFKLNLLETKCHVLDPTP VENCAVRQQHNHAVEMDCNVRIIHDIATFEDEVFVKCKSTPDSV ENVRRNCPKCPILLPPNDPHVVDSVEYVLNKHNEKLSGHVYEVL EISRGQHKYEPEAFYVEFAIVEVNCTAQEARDGHHQCHPYTAGE DHIAFCRATVFRSHASLEKPKDENFESDCVILDVKEGHAHSHLIQ QHIEKYSTSPGHNSTDEYVVECPVAFVEKEVPTDMSDHDTPPVK GCPGRVLHFQL*

B.jar_FETUA-1	*Bothrops jararaca*	genomic DNA	JAGTXL010000047.1	MNSLVALVLLGQIIGSTLSHHLQSHVDCNGEDAEKWAHMAVHYI<br>NEHNQHGYKCALNVINEIRLLPRRPHGTIVFLKLKVLETECHVLDP<br>TPTENCTVRPQHYHAVGGDCDVKIIHEEGGDKVIGAKCYSDPDS<br>VEDVRRNCPKCPILLNLNDPQVVDSVEYVLNKHNEKVSGHVYEV<br>LEISRGQHKNEPEAYYVEFAIVEVNCTAQEAHDDHHQCHPNTAG<br>ENHIGFCRATVFRSHASLEKPKDEQFESDCVIFDVKDGHAHSHLI<br>EHHVGKYSTSPGHNNTVLNLVHSHNHTSASHESHSHEHVTEVP<br>VAVAKREVPKDVPHDHTHPVKLCPGKVHHFEL*
B.jar_FETUA-2/<br>BJ46a	*Bothrops jararaca*	genomic DNA	JAGTXL010000047.1	MNSLVALVLLGQIIGSTLSSQVRGDLECDEKDAKEWTDTGVRYIN<br>EHKLHGYKYALNVIKNIVVVPWDGDWVAVFLKLNLLETECHVLDP<br>TPVKNCTVRPQHNHAVEMDCDVKIMFNVDTFKEDVFAKCHSTP<br>DSVENVRRNCPKCPILLPSNNPQVVDSVEYVLNKHNEKLSDHVY<br>EVLEISRGQHKYEPEAYYVEFAIVEVNCTAQELHDDHHHCHPNT<br>AGEDHIAFCRATVFRSHASLEKPKDEQFESDCVILHVKEGHAHS<br>HLIQQHVEKDSISPEHNNTALNFVHPHNDTSTSHESHEHLAEVPV<br>AFVKKELPKDISDRHTTPVKGCPGKVHHFEL*
B.jar_FETUA-3	*Bothrops jararaca*	genomic DNA	JAGTXL010000047.1	MNSMVALVLLGQIIGSTLSSQVRGDLPCDDEDSKWWADVGVRYI<br>NEHKLHGYKYALSVIKNIVVVPWDGDWVAVFLKLNLLETECHVLD<br>PTPVKNCTVRTQHNHAVEMDCDVKIMFNVETFKEDVFAKCHSTP<br>DSVENVRRNCPKCPILLPSNNPQVVDSVEYVLNKHNEQLSDHVY<br>EVLEISRGQHKYEPEAYYVEFAIVEVNCTAQEAHDDHHQCHPNT<br>AGEDHIGFCRATVFRSHASLEKPKDEQFESDCVILHVKKGHAHS<br>HLIQQHVEKDSISPEHNNTALNFVHPHNDTRASHESHEHLAKVPV<br>AFVKKELPKDISDRHTTPVVGCPGLRAVGQPY*
B.jar_FETUA	*Bothrops jararaca*	genomic DNA	JAGTXL010018706.1	MNSLVALVLLGQIIGSTHSYLVKEEDLNCNSKAAKYRADQAVHYI<br>NEHKLHGYKYALNVIKIFHLLPPEEFSTVFYLKLNLLETECHVFDP<br>TPVENCNVRPQHNHAVEMDCDVKIIFNFQFFKTEVFVKCNSTPD<br>SVENVRRNSPKCSILLPPNDPQVIDSVEYVLNKHNEQLSGHVYK<br>VLEISRGQHKYEPEAYYVEFAIMEINCTAQEAHDDHHQCHPNTA<br>GEDHIGFCRATVFRSHASLEKPKDEQFESDCVILDVKEGHAHSH<br>LIQQHVEKYSTSPGHNNTALNLVHLHNDTSTSHKSHSGKCPVVF<br>PKKKVPTDIPHHTHPVEGCPGKVLHFEL*

C.tig_FETUA-1	*Crotalus tigris*	genomic DNA	XP_039207926.1	MNSLVALVLLGQIIGCTFSHHLQSQVDCNGEDAEKWADMAVHYI NEHNLHGYKYTTNVINEIHVLPRRPHGVIVFLELKVLETQCHVLDP TPVENCTVRPQHYHAVGGDCDVKILHDEGVDKVIGAKCHSDPDS VEDVRQNCSKCPILLSLSDPHVVDSVEYVLNKYNEKLSGHVYEV LEISRGQHKYEPEAFYVEFAIVEVNCTAEEAHDDHHHCHPNTDG ENHIGFCKATVFRSHASLEKPKDEMFESDCVIFDVKEGHAHSHLI EHHVGKYSTSPGHNSTVLNLVHSHNHTSASHESHSHEHVMEVP VAVAKREVPIDVPHDHTHPVKLCPGKVHHFQL*
C.tig_FETUA-2	*Crotalus tigris*	genomic DNA	XP_039207927.1	MNSLVALVLLGXSIGSTLSSQVRGDLECDNKEAKEWADMAVRYI NEHKLHGYKQALNVIKNILVVPWDGDLVAVYLKLNLLETECHMLD PHPVENCTIRPQNNHAVKMDCNAKIMFDVVTFKQDVFVKCHSTP DSVENVRRNCPKCPILLPWNDSHVVDSVEYVLNKHNEKLSGHVY EVLEISRGQHKYEPEAFYVEFAIVEVNCTAEEAHDDHHHCHPNT AGEDHIGFCRATVFRSHASLEKPKDEKFESDCVILHVKEGYAYSH LLYKQIEKYNVPPEFRNTVLNLTHSHNHTSTSHESHEHVAEVPVA FVKKELPTDISDHDTPPVEGCPGKALHFQL*
C.tig_FETUA-3	*Crotalus tigris*	genomic DNA	XP_039207923.1	MNSLVALVLLGQIIGSTLSFQLAPNMDCNTKGTKDWADIGVRYIN EHKLHGYKQALNIIKIFRLLPSDGLSVMFHFKLNLLETECHVLDPT PVENCIVRQKNNHAVEMDCNVRIIHDIATFGDEVFVKCNSTPDSV ENVRRNCPKCPILLPPNDPHVVDSVEYVLNKHNEQLSGHVYEVL EISRGQHKYEPEAFYVEFAIVEVNCTAEEAHDDHHHCHPNTAGE DHIGFCRATVFRSHASLEKPKDEKFESDCVILHVKKGHAYSHLIE HHVGKYNTSPGRNSTDECVVECPVAFVNKEVPKDISDHDTPPVE GCPGKALHFQL*
C.tig_FETUA-4	*Crotalus tigris*	genomic DNA	XP_039207924.1	MNSLVVLVLLGQIIGSTHSYMVREEHLNCNSKAAKYRADQAVRYI NEHKLHGYKQALNIIKTFHLLPPERFTTVFYLKLNLLETECHVLDP TPVENCTVRPQNNHAVEMDCDVKINFNFEFFKTEIFVKCNSTPNS VENVRRNCPECPILLPPNDPHVIDSVEYVLNKHNEKLSGHVYEVL EISRGQHKYEPEAFYVEFAIVEVNCTAEEAHDDHHHCHPNTAGE DHIGFCRATVFRSHASLEKPKDEKFESDCVILHVKEGHATSHLIE HHTGKYSTSPGRNSTDECVVECPVAFVKKEVPTHIPHHTHPVEG CPGKVFHFQL*

C.tig_FETUA-5	*Crotalus tigris*	genomic DNA	LOC120311907	MNSLVVLVLLGQIIGSTLSYMVREEDLNCNSKAAKYRADQAVHYI<br>NEHNLHGYKQALNIIKTIRLLPSDGLSVIIYLKLNLLETECHVLDPTP<br>VENCTVRQQNNHAVETDCDVKIIYNIETFKDEFLVKCHSTPGSVE<br>NILQDCPKCPILMPLSEPHVTDSVEYVLNKHNEQLSGHVYEVLEI<br>SRGQHKYEPEAFYVEFAIVEVNCTAQEAHDDHHHCHPNTDGEN<br>HIGFCRATVFRSYASLEKPKVEMSESDCVILDVKEGHAHSHLIEH<br>HVGKYSISPGHNNTVLNLAHSHNHTSASHESHSHEHVTEVPVAV<br>AKREVPTDIPHHHTPSVDGCPGKVLHFKL*
C.hor_FETUA-5	*Crotalus horridus*	genomic DNA	OP223732	MNSLVALVLLGQIIGSTHSYMVREEDLNCNSKAAKYRADQAVHYI<br>NEHNLHGYKQALNIIKTIRLLPSDGLSVIMYLKLNLLETECHVLDPT<br>PVENCTVRQQHNHAVETDCDVKIIYNIETFKDEFLVKCHSTPGSV<br>EKILEDCPKCPLLLSLSDPHVVDSVEYVLNKHNEKLSGHVYEVLEI<br>SRGQHKYEPEAFYVEFAIVEVNCTAQEAHDDHHHCHPNTAGEN<br>HIAFCRATVFRSYASLEKPKDEKFESDCVILHVKEGHTHSHLIEHH<br>IGKYSTSPGHNSTVLNLAHSHNHTSASHESHSHEHVTEVPVAVA<br>KREDPTDIPHHHTPSVDGCPGKVLHFKL*
C.hor_FETUA-4	*Crotalus horridus*	genomic DNA	OP223732	MNSLVVLVLLCQIIGSTYSYMVREEDLNCNSKAAKYRADQAVRYI<br>NEHKLHGYKQALNIIKIFHLLPPERFMTVFYLKLNLLETECHVLDP<br>TPVENCTVRPQNNHAVEMDCDVKINFNFEFFKTEIFVKCNSTPDS<br>VENVQRNCPECPILLPPNDPHVINSVEYVLNKHNEKLSGHVYEVL<br>EISRGQHKYEPEAFYVEFAIVEVNCTAEEAHNDHHHCHPNTAGK<br>DHIGFCRATVFRSHASLEKPKDEMFESDCVIFDVKKGHAHSHLIQ<br>QHIGKYNISPGHNNAVLNLAHSHNDTRASQESHSDECVVECPVA<br>FVKKEVPTHIPHHTHPVEGCPGKVFHFQL*
C.hor_FETUA-3	*Crotalus horridus*	genomic DNA	OP223732	MNSLVALMLLGQIIGSTLSFQLAANMDCNTKGTKDWADIGVRYIN<br>EHKLHGYKQDLNIIKIFRLLPSDGRSVIFHFKLNLLETECHVLDPTP<br>VENCTVRQQNNHAVEMDCNVRIIHDIATFEDEVFVKCNSTPDSV<br>ENVRRNCPKCPILLPPNDPHVVDSVEYVLNKHNEKLSGHIYEVLE<br>ISRGQHKYEPEAFYVEFAIVEVNCTAEEAHDDHHHCHPNTDGEN<br>HIGFCRSTVFRSHASLEKPKDEKFESDCVILHVKEGHAHSHLIEH<br>HVGKYSTSPGRNSTDECVVECPVAFVNKEVPKDISDHDTPPVEG<br>CPGKVLHFQL*
C.hor_FETUA-2	*Crotalus horridus*	genomic DNA	OP223732	MNSLVALVLLGQIIGSTLSSQVRGDLECDDKEAKEWADIAVRYIN<br>EHKLHGYKQALNVIKNILVVPWNGDLVAVFLKLNLLETECHVLDP<br>TPVENCTVRPQNNHAVEMDCDAKIMFDVVTFKTDVFVKCHSTPD<br>SVENVRRNCPKCPILLPRNDSHVVDSVEYVLNKHNEKLSGHVYE<br>VLEISRGQHKSEPEAFYVEFAIVEVNCTAQEAHDDHHHCHPNTT

				GEDHIGFCRATVFRSHASLEKPKDEKFESDCVIFDVKEGHAHSHL IEHHVGKYSTSPGRNSTDECVVECPVAFVNKELPTDISDHHTTPV KGCPGKVLHFQL*
C.hor_FETUA-1	*Crotalus horridus*	genomic DNA	OP223732	MNSLVALVLLGQIIGCTFSHHLQSQVDCNGEDAEKWADMAVHYI NEHNLHGYKYTNNVINEIHVLPRRPRGEIVFLELKVLETQCHVLD PTPVENCTVRPLHYHAVGGDCDVKIIHDEGVDKVIGAKCHSDPD SVEEVRRNCPECPILLSLSDPHVINCVEYVLNKYNEKLSGHVYEV LEISRGQHKYEPEAFYVEFAIVEVNCTAEEAHDDHHHCHPNTDG ENHIGFCRATVFRSHASLEKPKDEMFESDCVIFDVKEGHAHSHLI EHHVGKYSTSPGHNNTVLNLIHSHNHTSASHESHSHEHMTEVPV AVAKREVPTDVPHDHIHPVKLCPGKVHHFKL*
C.scu_FETUA-5	*Crotalus scutulatus*	genomic DNA	OP566391	MNSLVALVLLGQIIGSTHSYMVREEDLNCNSKAAKYRADQAVHYI NEHNLHGYKQALNIIKTIRLLPSDGLSVIIYLKLNLLETECHVLDPTP VENCTVRQQNNHAVETDCDVKIIYNIETFKDEFLVKCHSTPGSVE KILQDCPKCPILLSLSDPHVVDSVEYVLNKHNEQLSGHVYEVLEIS RGQHKYEPEAFYVEFAIVEVNCTAQEAHDDHHHCHPNTAGEDHI AFCRATVFRSYASLEKPKDEMSESDCVILDVKEGHAHSHLIEHHT GKYSTSPGHNSTVLNLAHSHNHTSASHESHSHEHVTEVPVAVAK REVPTDIPHHHTPSVDGCPGKVLHFKL*
C.scu_FETUA-4	*Crotalus scutulatus*	genomic DNA	OP566391	MNSLVALVLLGQIIGSTLSYMVREEDLNCNSKAAKYRADQAVRYI NEHKLHGYKQALNIIKTFHLLPPERFTTVFYLKLNLLETECHVLDP TPVENCTVRPQYNHAVEMDCDVKINFNFEFFKTEIFVKCNSTPDS VENVR*NCPECPILLPPNDPHVIDSVEYVLNKHNEKLSGHIYEVLE ISRGQHKYEPEAFYVEFAIVEVNCTAEEAHDDHHHCHPNTAGED HIGFCRATVFRSHASLEKPKDEKFESDCVILHVKKGHAHSHLIQQ HIGKYNISPGHHNTVLNLAHSHNDTRASQESHSDECVVECPVAF VKKEVPTHIPHHTHPVEGCPGKVHHFKL*
C.scu_FETUA-3	*Crotalus scutulatus*	genomic DNA	OP566391	DGLSVIFHFKLNLLETECHVLDPTPLENCTVRQQNNHAVEMDCN VRIIHDIATFEDEVFVKCNSTPDSVENVRRNCPKCPILLPPNDPHV VDSVEYVLNKHNEKLSGHIYEVLEISRGQHKYEPEAFYVEFAIVE VNCTAEEAHDDHHHCHPNTDGENHIGFCRATVFRSHASLEKPK DEKFESDCVILHVKKGHAYSHLIEHHVGKYNTSPGRNSTDECVV ECPVAFVNKEVPKDISDHDTPPVEGCPGKALHFQL*

C.scu_FETUA-2	*Crotalus scutulatus*	genomic DNA	OP440564	MNSLVALVLLGQIIGSTLSSQVRGDLECDDKEAKEWADMAVRYINEHKLHGYKQALNVIKNILVVPWDGDLVAVYLKLNLLETECHVLDPTPVENCTIRPQNNHAVKMDCNAKIMFDVVTFKQDVFVKCHSTPDSVENVRRNCPECPILLPRNDSHVVDSVEYVLNKHNEKLSGHVYEVLEISRGQHKYEPEAFYVEFAIVEVNCTAQEAHDDHHHCHPNTAGENHIGFCRATVFRSHASLEKPKDEKFESDCVILHVKEGHAHSHLIEHHVGKYNTSPGRNSTDECVVESSLLTKATGRPVAFVNKEVPADISDHHTTPVKGCPGKVLHFQL*
C.scu_FETUA-1	*Crotalus scutulatus*	genomic DNA	OP440564	MNSLVALVLLGQIIGCTFSHHLQSQVDCNGEDAEKWADMAVHYINEHNLHGYKYTTNVINEIHVLPRRPHGEIIFLELKVLETQCHVLDPTPVENCTVRPLHYHAVGGDCDVKILHDEGVDKVIGAKCHSDPDSVEDVRRNCPNCPILLSLSDPHVVDSVEYVLNKHNEKLSGHVYEVLEISRGQHKYEPEAFYVEFAIVEVNCTAEEAHDDHHHCHPNTAGENHIGFCRATVFRSHASLEKPKDEKFESDCVIFDVKEGHAHSHLIEHHTGKYSTSPGHNNTVLNLVHSHNHTSASHESHSHEHMTEVPVAVAKREVPTDVPHDHTHPVKLCPGKVHHFKL*
C.atx_FETUA-5	*Crotalus atrox*	cDNA	OP616715	MNSLVALVLLGQIIGSTHSYMVREEDLNCNSKAAKYRADQAVRYINEHNLHGYKQALNIIKTIRLLPSDGLLVVIYLKLNLLETECHVLDPTPVENCTVRQQNNHAVETDCDVKVIYNIETFKDEFLVKCHSTPGSVEKILQDCPKCPILLSLSDPHVVDSVEYVLNKHNEQLSGHVYEVLEISRGQHKYEPEAFYVEFAIVEVNCTAQEAHDDHHHCHPNTAGENHIGFCRATVFRSHASLEKPKDEMSESDCVILDVKEGHAHSHLIEHHTGKHSTSPGHNSTVLNLAHSHNHTSASHESHSHEHVMEVPVAVANREDPTDIPHHHTPSVDGCPGKALHFQL*
C.atx_FETUA-4	*Crotalus atrox*	cDNA	OP616716	MNSLVALVLLGQIIGSTLSYMVKEEDMNCNSKAAKYRADQAVRYINEHKLHGYKQALNIIKTFHLLPPERFTTVFYLKLNLLETECHVLDPTPVENCTVRPQNNHAVEMDCDVKINFNFEFFKTEIFVKCNSTPDSVENVQRNCPKCPILLPPNDPHVINSVEYMLNKHNEKLSGHVYEVLEISRGQHKYEPEAFYVEFAIVEVNCTAEEAHDDHHHCRPNTAGEDHIGFCRSTVFRSHASLEKPKDEMFESDCVILHVKKGHSHSHLIQQHIGKYNISPGHHNTVLNLAHSHNDTRASQESHSDECVVECPVAFVKKEVPTHIPHHTHPVEGCPGKVHHFKL*

C.atx_FETUA-3	*Crotalus atrox*	cDNA	OP616717	MNSLVALVLLGQIIGSTLSFQLAGNMDCNTKGTKDWADIGVRYIN<br>EHKLHGYKQALNIIKIFRLLPSDGRSVIFHFKLNLLETECHVLDPTP<br>LENCTVRQQNNHAVEMDCNVRIIHDIATFEDEVFVKCNSTPDSVE<br>NVRRNCPKCPILLPPNDPHVIDSVEYVLNKHNEKLSGHIYEVLEIS<br>RGQHKYEPEAFYVEFAIVEVNCTAEEAHDDHHDCHPNTDGENHI<br>GFCRATVFRSHASLEKPKDEKFESDCVILHVKKGHAYSHLIEHHV<br>GKYNTSPGRNSTDECVVQCPVAFVNKEVPKDISDHDTPPVEGC<br>PGKALHFQL*
C.atx_FETUA-2	*Crotalus atrox*	cDNA	OP616718	MNSLVALVLLGQIIGSTLSSQVRGDLECDDKEAKEWADMAVRYI<br>NEHKLHGYKQALNVIKNILVVPWDGDLVAVYLKLNLLETECHVLD<br>PHPVENCTIRPQNNHAVKMDCNAKIMFDVVT<br>FKQDVFVKCHSTPDSVENIRRNCPKCPILLPRNDSHVVDSVEYVL<br>NKHNEKLSGHIYEVLEISRGQHKYEPEAFYVEFAIVEVNCTAQEA<br>HDDHHHCHPNTAGEDHIGFCRATVFRSHASLEKPKDEKFESDCV<br>ILHVKEGHVHSHLIEHHTGKYSTSPGSNSTDECVVQCPVAFVNK<br>ELPTDISDHHTTPVKGCPGKVFHFQL*
C.atx_FETUA-1	*Crotalus atrox*	cDNA	OP616719	MNSLVALVLLGQIIGCTFSHHLQSQIDCNGEDAEKWADMAVHYIN<br>EHNLHGYKYTTNVINEIHVLPWRPHGEIIFLELKVLETQCHVLDPT<br>PVENCTVRPLHYHAVGGDCDVKIIHDEGVDKVIGAKCHSDPDSV<br>ENVRQNCPECPILLSLSDPHVVDCVEYVLNKYNEKLSGHIYEVLEI<br>SRGQHKYEPEAFYVEFAIVEVNCTAEEAHDDHHHCHPNTAGEN<br>HIGFCRATVFRSHASLEKPKDEMFESDCVIFDVKEGHAHSHLIEH<br>HTGKYSTSPGHNNTVLNLIHSHNHTSASHESHSDEHMTEVPVAV<br>AKREVPTDVPHDHTHPVKLCPGKVHHFKL*
C.ada_FETUA-5	*Crotalus adamanteus*	genomic DNA	OP223730	MNSLVALVLLGQIIGSIHSYMVREEDLNCNSKAAKYRADQAVQYI<br>NEHNLHGYKQALNIIKTIRLLPSDGLLVVIYLKLNLLETECHVLDPT<br>PVENCTVRPQNNHAVETDCDVKIIYNIETFKDEFLVKCHSTPGSV<br>EKILQDCPKCPILLPPNDPHVVDVEYVLNKHNEKLSGHVYEVLEIS<br>RGQHKYEPEAFYVEFAIVEVNCTAQEAHDDHHHCHPNIAGEDHI<br>GFCRATVFRSYASLEKPKDEKFESDCVILDVKEGHAHSHLIQQHI<br>GKYSTSPGHNNTVLNLAHSHNHTSASHESHSHEHVTEVPVAVA<br>KREDPTDIPHHHTPSVDGCPGKVLHFKL*

C.ada_FETUA-4	*Crotalus adamanteus*	genomic DNA	OP223730	MNSLVVLVLLGQIIGSTHSYMVREEDMNCNSKAAKYRADQAVRYINEHKLHGYKQALNIIKTFHLLPPERFTTVFYLKLNLLETECHVLDPTPVENCTVRPQNNHAVEMDCDVKINFNFEFFKTEIFVKCNSTPDSVENVRRNCPECPILLPPNDPHVIDSVEYVLNKHNEKLSGHIYEVLEISRGQHKYEPEAFYVEFAIVEVNCTAEEAHDDHHHCHPNTDGENHIGFCRATVFRSHASLEKPKDEKFESDCVILHVKEGHAHSHLIQQHIGKYNISPGHNNTVLNLAHSHNDTRASQESHSDECVVECPVVFVKKEVPTHIPHHTHPVEGCPGKVFHFQL*
C.ada_FETUA-3	*Crotalus adamanteus*	genomic DNA	OP223730	MNSLVALMLLGQIIGSTLSFQLAGNMDCNTKGTKDWADIGVRYINEHKLHGYKQALNIIKIFRLLPSGGLSVIFHFKLNLLETECHVLDPTPVENCTVRPQNNHAVEMDCNVRIIHDIATFEDEVFVKCNSTPDSLENVRRNCPKCPILLPPNDPHVVDSVEYVLNKHNEKLSGHIYEVLEISRGQHKYEPEAFYVEFAIVEVNCTAEEAHDDHHHCHPNTDGENHIGFCRATVFRSHASLEKPKDEKFESDCVILHVKEGHAHSHLIQHHTGKYSTSPGRNSTDECVVECPVAFVNKELPTDISDHDTPPVEGCPGKVLHFQL*
C.ada_FETUA-2	*Crotalus adamanteus*	genomic DNA	OP223730	MNSLVALVFLGQIIGSTLSSQVRGDLECDDKEAKEWADMAVRYINEHKLHGYKEALNVIKNILVVPWNGDLVAVFLKLNLLETECHVLDPTPVENCTVRPQNNHAVEMDCDAKIMFDVVTFKKDVFVKCLSTPDSVENVRRNCPKCPILLPRNDSHVVDSVEYVLNKHNEKLSGHVYEVLEISRGQHKYEPEAFYVEFAIVEVNCTAQEAHDDHHHCHPNTAGEDHIGFCRATVFRSHASLEKPKDEKFESDCVILHVKEGHAHSHLIEHHTGKYSTSPGRNSTDECVVQCPVAFVNKELPTDISDHHTTPVKGCPGKVFHFQL*
C.ada_FETUA-1	*Crotalus adamanteus*	genomic DNA	OP223731	MNSLVALVLLGQIIGCTFSHHLQSQIDCNGEDAEKWADMAVHYINEHNLHGYKYTTNVINEIHVLPRRPHGEIIFLELKVLETQCHVLDPTPVENCTVRPLHYHAVGGDCDVKIIHDEGVDKVIGAKCHSDPDSVENVRQNCPECPILLSLSDPHVIDSVEYVLNKYNEKLSGHVYEVLEISRGQHKYEPEAFYVEFAIVEVNCTAEEAHDDHHHCHPNTDGENHIGFCRATVFRSHATLEKPKDEMFELDCVIFDVKEGHAHSHLIEHHVGKYSTSPGHNNTVLNLIHSHNHTSASHESHSHEHMMEVPVAVAKREVPTDVPHDHTHPVKLCPGKVHHFKL*

SI References

1. N. L. Dowell, *et al.*, The Deep Origin and Recent Loss of Venom Toxin Genes in Rattlesnakes. *Current Biology* **26**, 2434–2445 (2016).
2. C.-S. Chin, *et al.*, Nonhybrid, finished microbial genome assemblies from long-read SMRT sequencing data. *Nat Methods* **10**, 563–569 (2013).
3. N. L. Dowell, *et al.*, Extremely Divergent Haplotypes in Two Toxin Gene Complexes Encode Alternative Venom Types within Rattlesnake Species. *Current Biology* **28**, 1016-1026.e4 (2018).
4. S. M. Kiełbasa, R. Wan, K. Sato, P. Horton, M. C. Frith, Adaptive seeds tame genomic sequence comparison. *Genome Res* **21**, 487–493 (2011).
5. S. F. Altschul, W. Gish, W. Miller, E. W. Myers, D. J. Lipman, Basic local alignment search tool. *J Mol Biol* **215**, 403–410 (1990).
6. H. Li, "Aligning sequence reads, clone sequences and assembly contigs with BWA-MEM" (2013).
7. H. Li, J. Barrett, A statistical framework for SNP calling, mutation discovery, association mapping and population genetical parameter estimation from sequencing data. **27**, 2987–2993 (2011).
8. P. Danecek, *et al.*, Twelve years of SAMtools and BCFtools. *Gigascience* **10**, giab008 (2021).
9. F. Hahne, R. Ivanek, "Visualizing Genomic Data Using Gviz and Bioconductor" in *Statistical Genomics: Methods and Protocols*, S. D. Ewy Mathé, Ed. (2016), pp. 335–351.
10. H. F. Williams, *et al.*, Mechanisms underpinning the permanent muscle damage induced by snake venom metalloprotease. *PLoS Negl Trop Dis* **13** (2019).
11. A. I. Nesvizhskii, A. Keller, E. Kolker, R. Aebersold, A Statistical Model for Identifying Proteins by Tandem Mass Spectrometry. *Anal Chem* **75**, 4646–4658 (2003).
